# Evaluation of cold tolerance in sorghum germplasm from the Chishui River Basin in China: insights from germination, field trials, and physiological assays

**DOI:** 10.3389/fpls.2025.1630271

**Published:** 2025-09-02

**Authors:** Hongli Yang, Wenxue Cui, Yue Liu, Wen Zhang, Jiangli Liu, Qing Liu, Hangmei Yao, Yue Huang, Jiadai Tang, Kaixian Wu

**Affiliations:** ^1^ College of Brewing Engineering, Moutai Institute, Renhuai, Guizhou, China; ^2^ College of Resources and Environment, Moutai Institute, Renhuai, Guizhou, China; ^3^ Guizhou Institute of Prataculture, Guizhou Academy of Agricultural Science, Guiyang, Guizhou, China

**Keywords:** germplasm resources, low temperature, cold tolerance assessment, sorghum, Chishui River Basin

## Abstract

**Introduction:**

Exploring cold-tolerant sorghum germplasm is crucial for improving production in low-temperatures regions. However, the cold tolerance of local sorghum germplasms in the Chishui River Basin, located in the southwestern China, remains poorly characterized.

**Methods:**

We evaluated 71 sorghum germplasms at Maotai Institute from 2022 to 2024 using germination tests, pot trials, and field experiments with a two-factor design. Germination potential, germination percentage, and seedling vigor traits (plumule and radicle length and weight) varied significantly among the 71 sorghum germplasm (*P* < 0.01). Membership function method identified several highly cold-tolerant accessions (e.g., Nos. 12, 22) and cold-sensitive ones (Nos. 17, 44), establishing 15 °C as an optimal temperature for germination stage cold tolerance assessment.

**Results:**

Cluster analysis classified these into four groups: cold-tolerant (11), moderately cold-tolerant (22), moderately cold-sensitive (29), and cold-sensitive (9). Agronomic data collected under two early sowing conditions (severe and mild low-temperature stress) demonstrated that several germplasm accessions, like No. 12, maintained high emergence rates (97% and 100% *VS*. 100% in conventional sowing) and grain weight per panicle (63.3 g and 53.4 g *VS*. 45.9 g in conventional sowing) without significant reductions, whereas others, such as Nos. 17 and 48, showed marked decreases (*P* < 0.01). The superior cold tolerance of accessions Nos. 2, 12, and 22 was confirmed through membership function analysis (D-value > 0.6). A significant positive correlation between comprehensive cold tolerance ratings at both the germination and field stages was observed (*r* = 0.687, *P* < 0.05). Cold-tolerant germplasms such as No. 12 exhibited high cold tolerance coefficients for chlorophyll content (CHL: 0.98), relative water content (RWC: 0.99), superoxide dismutase (SOD: 1.77), and peroxidase (POD: 2.03), and low malondialdehyde (MDA: 1.20), indicating enhanced membrane stability and oxidative stress tolerance (*P* < 0.05). Stepwise regression highlighted a strong correlation (*r* = 0.976, *P* < 0.01) between predicted values and field D-values, identifying SOD and POD activities as critical physiological indicators of cold tolerance.

**Conclusion:**

This study not only identifies valuable cold-tolerant sorghum germplasms but also elucidates their physiological mechanisms, providing essential insights and materials for developing cold-tolerant varieties and resilient cultivation practices in the Chishui River Basin.

## 1 Introduction

Low-temperature stress is a critical abiotic factor limiting crop productivity, particularly affecting C4 plants like sorghum (*Sorghum bicolor* (L.) Moench), which are adapted to warm and arid climates (Paterson et al., 2009; Aregawi et al., 2022). Sorghum, originating from the tropical and subtropical regions of Africa, thrives in high temperatures and humid conditions (Hariprasanna and Patil, 2015). Despite its preference for these environments, sorghum displays remarkable ecological adaptability, capable of withstanding drought, low temperatures, poor soils, and salinity. It is cultivated globally and ranks as the fifth most important cereal crop worldwide, following maize, wheat, rice, and barley (Habyarimana et al., 2022). The versatility of sorghum makes it an indispensable resource for food, feed, biofuel, industrial starch, and brewing applications (Aregawi et al., 2022; Paterson et al., 2009). However, significant evidence indicates that sorghum yields are severely affected by cold stress (Yu et al., 2004; Maulana and Tesso, 2013). Enhancing the cold tolerance of sorghum is therefore crucial for improving its productivity. By enhancing its cold resistance, we can expand the cultivation range of sorghum, thereby leveraging its strong ecological adaptability to address challenges posed by global climate change, diminishing water resources, and declining soil quality (Parra-Londono et al., 2018).

The development and breeding of cold-tolerant sorghum varieties are pivotal strategies for enhancing productivity in low-temperature environments. Internationally, efforts to evaluate and explore sorghum germplasm for cold tolerance have gained momentum, with notable initiatives in Germany (Bekele et al., 2014) and the United States (Ortiz et al., 2017). In China, significant attention has been directed towards the collection, evaluation, and utilization of sorghum germplasm resources (Ding et al., 2024; Lu and Dahlberg, 2001), yet research specifically addressing cold tolerance remains limited. China harbors a rich diversity of sorghum germplasm, particularly within the Chishui River Basin in southwestern China (Yan et al., 2018). This region is renowned as the heartland of Chinese baijiu production, with over 80% of the nation’s sorghum dedicated to brewing purposes. Consequently, the Chishui River Basin emerges as one of the primary sorghum cultivation zones in China. A critical challenge in this area is the frequent occurrence of late spring cold spells during crucial sowing periods, where temperatures can plummet below 10 °C (Wang et al., 2012), significantly impacting sorghum growth. Enhancing the cold tolerance of brewing sorghum is therefore essential for ensuring robust production of specialized sorghum required for the core production areas of sauce-flavored baijiu.

Beyond mitigating the direct impacts of late spring cold spells and other low-temperature events, evaluating and selecting cold-tolerant sorghum germplasm in the Chishui River Basin holds significant potential for promoting sorghum and baijiu production beyond this region. The scale of baijiu production in the Chishui River Basin is expanding continuously, with projections indicating it will exceed 50 million hectoliters by 2028. Consequently, the demand for high-quality brewing sorghum is expected to rise substantially. However, arable land within the Chishui River Basin is limited. Ensuring a sustainable supply of premium brewing materials necessitates extending sorghum cultivation to adjacent regions with similar climatic and ecological conditions. The areas surrounding the Chishui River Basin are located on the Yunnan-Guizhou Plateau, which is characterized as the only plateau region in China without extensive plains, with an average elevation of approximately 1,110 meters. Notably, upstream areas of the Chishui River Basin often exceed 1,200 meters in altitude, where lower temperatures pose potential cold stress challenges. These higher altitudes provide both opportunities and challenges for sorghum cultivation, requiring tailored strategies for adaptation.

Cold tolerance in sorghum can be assessed through multiple approaches. Initial stages of germination and seedling growth are particularly sensitive to low temperatures (Bekele et al., 2014), making evaluations under cold stress conditions crucial for understanding early-stage cold tolerance. However, a comprehensive assessment must also include field-based evaluations (Yu et al., 2004) to capture the full spectrum of environmental interactions. Additionally, physiological and biochemical responses to cold stress not only illuminate underlying mechanisms but also provide effective means for validating cold tolerance across different germplasm resources. Despite these insights, most existing studies have been conducted under controlled environments (Ortiz et al., 2017; Yu et al., 2004), with limited validation under natural field conditions (Franks et al., 2006; Maulana and Tesso, 2013). In China, particularly within the Chishui River Basin in the southwestern region, local sorghum germplasm exhibits rich genetic diversity (Yan et al., 2018). However, their potential for cold tolerance and resource exploration has received scant attention. Given the expanding demand for high-quality brewing sorghum in this region, it is imperative to conduct systematic evaluations of cold tolerance by integrating assessments of germination performance, field performance, and physiological responses.

The cold tolerance of brewing sorghum germplasm in the Chishui River Basin is still poorly understood. This study aimed to assess its potential as a genetic resource for breeding cold-tolerant varieties and developing strategies to counter low-temperature stress. Such improvements are essential for enhancing productivity in regions frequently affected by chilling or frost. A total of 71 germplasm accessions from the basin were evaluated for cold tolerance using an integrated approach. This included laboratory germination assays, field performance monitoring, and physiological assessments under controlled pot-culture conditions. These germplasm accessions have been traditionally cultivated in mountainous areas under diverse climatic and edaphic conditions. Their long-term cultivation history suggests adaptation to abiotic stresses. We therefore hypothesize that this germplasm collection harbors substantial genetic variation in cold tolerance, with certain genotypes exhibiting pronounced resilience to low-temperature stress.

## 2 Materials and methods

### 2.1 Materials

This study evaluated a total of 71 sorghum accessions, including 56 germplasm materials sourced from the midstream and upstream regions of the Chishui River Basin (comprising variants of sorghum cultivars used for Moutai baijiu production), 11 local sorghum resources collected from outside the Chishui River Basin in Guizhou Province, and 4 commercially cultivated sorghum varieties predominant in the Chishui River Basin. Detailed information on the names, origins, and types of these resources and varieties is provided in **Supplementary Table S1**. Given that Hongyingzi glutinous sorghum is the preferred raw material for producing sauce-flavored baijiu in Guizhou, with the midstream and upstream areas of the Chishui River being its primary cultivation zones, the majority of the collected sorghum resources were variants of the Hongyingzi cultivar.

Sorghum was cultivated at the Sorghum Research Base of the Moutai Institute (coordinates: 27°7217,N, 106°303,E). Seeds were harvested at physiological maturity and dried in a laboratory dryer at 35°C until the moisture content reached below 12% (Salas Fernandez et al., 2014). After threshing, the seeds were stored in a seed storage cabinet (Hangzhou Top Instrument Co., Ltd., TP-DC50C) maintained at 6°C with a relative humidity of 30%. Prior to testing, seeds were manually sorted to remove any damaged or compromised seeds, ensuring that only healthy and intact seeds were used for experiments.

### 2.2 Experimental design and methods

#### 2.2.1 Cold tolerance test at the germination stage

Seed vigor under cold stress was evaluated according to the Germination Test of Crop Seed Inspection Regulations (GB/T 3543.4-1995) established by the Standardization Administration of China. The experiment was conducted in 2022 using an artificial climate chamber (Shanghai Yiheng Scientific Instruments Co., Ltd., MGC-350HP-2L). The experiment was conducted as a two-factors design, with temperature and germplasm materials as the experimental factors. Three temperature regimes were established: 10 °C for relatively extreme cold stress, 15 °C for moderate cold stress, and 25 °C as the control. Environmental conditions were set to 8 hours of light and 16 hours of darkness per day, with a relative humidity of 60% and a light intensity of 24,000 lx, following protocols from previous studies (Salas Fernandez et al., 2014; Yang et al., 2020b). Healthy and viable seeds (uniform size, no signs of mold) were selected and surface-sterilized by soaking in 70% ethanol for 1 minute, followed by treatment with 0.5% sodium hypochlorite for 10 minutes. Seeds were then rinsed at least three times with sterile water and dried before use. Each seed was placed in a 9 cm diameter Petri dish lined with double-layered filter paper, with 25 seeds per dish. Each dish received an equal amount of distilled water. Seeds were carefully positioned and marked, and each accession was replicated five times (five Petri dishes).

Seeds were incubated under the specified conditions for 10 days. Seedling emergence rate and seed vigor were assessed starting on the third day of cultivation, with sterile water replenished every two days. On the tenth day, five seedlings from each petri dish were sampled to evaluate eight parameters: germination potential (GPo, calculated as the number of germinated seeds on day 3 divided by the total number of tested seeds × 100%), germination percentage (GPe, calculated as the number of germinated seeds on day 10 divided by the total number of tested seeds × 100%), plumule length (PL, measured from the base of the plumule to the tip of the longest leaf using a ruler after carefully removing and drying the seedlings), radicle length (RL, measured from the base of the plant to the distal end of the radicle system using a ruler), plumule fresh weight (PFW, determined by cutting the seedlings at the radicle-plumule junction and weighing the plumule portion using an electronic balance), radicle fresh weight (RFW, determined by cutting the seedlings at the radicle-plumule junction and weighing the radicle portion using an electronic balance), radicle-to-plumule length ratio (calculated as radicle length divided by plumule length), and radicle-to-plumule fresh weight ratio (calculated as radicle fresh weight divided by plumule fresh weight). Germination was defined as the radicle reaching the length of the seed and the plumule reaching half the seed length. Each treatment was replicated five times.

#### 2.2.2 Cold tolerance test in field trials

**Figure 1:**
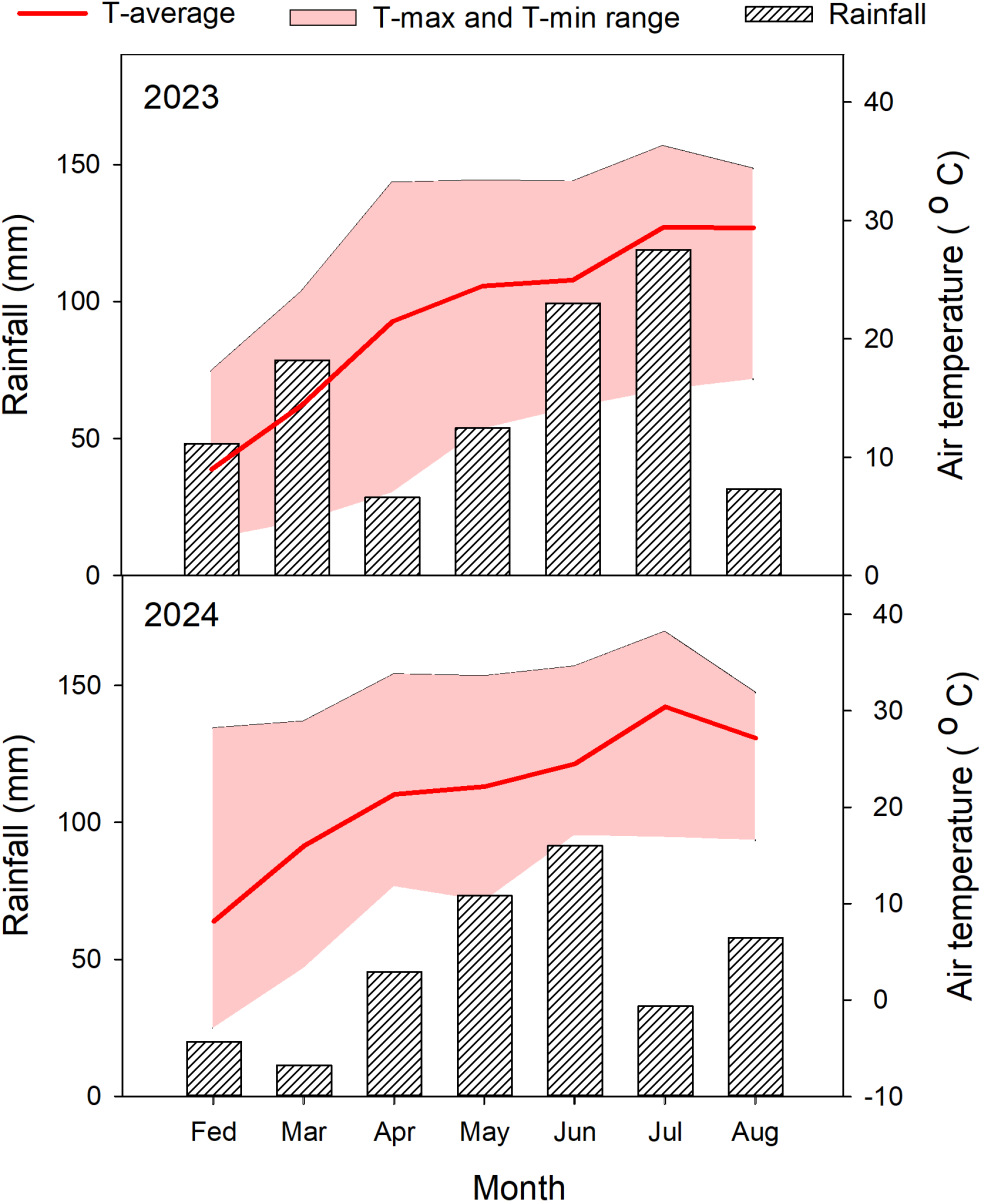
Monthly precipitation and temperature during the sorghum growing seasons in 2023 and 2024. T-average: average temperature. T-Max and T-min range: minimum and maximum temperature.

Based on the 2022 indoor germination screening, we selected 25 representative sorghum germplasm accessions spanning the full spectrum of observed cold tolerance, including 3 cold-tolerant, 19 intermediate, and 3 cold-sensitive types, for field validation. Field trials were conducted in 2023 and 2024 at the Sorghum Research Base of the Moutai Institute, located in the middle reaches of the Chishui River Basin, China, at an altitude of 840 m. The average temperature during the entire growth period was 19.05 °C, with total rainfall amounts of 458.61 mm in 2023 and 332.02 mm in 2024 (**Figure 1**). The experimental site is characterized by yellow soil (Chinese classification), with soil properties averaged over 2023 and 2024 as follows: pH 7.99, organic matter content 2.88 g/100g, total nitrogen 1.60 g/kg, total phosphorus 0.82 g/kg, total potassium 27.00 g/kg, available nitrogen 152.71 mg/kg, available phosphorus 32.90 mg/kg, and available potassium 89.28 mg/kg.

The experiment was conducted using a randomized complete block design with three replications. Three simulated temperature treatments were established by adjusting the sowing dates. The conventional sowing date (March 25, when the daily average temperature exceeds 15°C) served as the control. Two earlier sowing dates were set to impose cold stress: severe low-temperature early sowing (February 15, with daily average temperatures below 10°C, representing a stronger cold stress effect) and mild low-temperature early sowing (March 5, with daily average temperatures around 15°C, representing moderate cold stress). Each germplasm resource was planted in two rows, with eight planting holes per row, spaced 40 cm between plants and 70 cm between rows. Prior to sowing, the field was prepared, and 18 seeds were directly sown per hole along with fertilizer applied at a rate of 1.8 tons per hectare (containing ≥ 5.5% total nutrients N+P_2_O_5_+K_2_O, ≥ 50% organic matter, and ≤ 30% moisture content). Seeds were covered with 4 cm of soil. At the three-leaf stage, seedlings were thinned to two plants per hole, achieving a final planting density of 7,411 plants per hectare. No artificial irrigation was applied during the trial, and pest, disease, and weed management followed local organic farming practices. Seedling emergence was monitored daily from the first sowing date until no new seedlings emerged for three consecutive days across all resources. The emergence rate was calculated as: Emergence Rate (%)= Number of emerged seedlings / Total number of tested seeds × 100  (Salas Fernandez et al., 2014). At maturity, five representative plants per resource (or three if emergence rates were too low to provide five plants) were selected from each of the three sowing dates to measure single-panicle grain weight.

#### 2.2.3 Pot experiment on physiological cold tolerance

To investigate the physiological responses of different genetic materials to low temperatures and further validate their cold tolerance based on findings from the indoor germination test (Section 2.2.1) and field trial (Section 2.2.2), nine sorghum resources were selected: four cold-tolerant materials (12, 95, 2, 22), three intermediate materials (13, 3, 1), and two cold-sensitive materials (17, 15). Seeds of each resource were sown in pots under controlled conditions at 25°C using a uniform cultivation protocol. All pots utilized the same substrate mixture (peat: perlite = 3:1) and specifications. Each pot contained four plants, with 24 pots each resource. When the sorghum seedlings reached the 4–5 leaf stage, 12 randomly selected pots per resource were subjected to low-temperature stress treatment in a growth chamber maintained at 10°C, with a light intensity of 250-300 μmol m^-2^ s^-1^, a photoperiod of 12 hours, and relative humidity of 60%-70%. The remaining 12 pots were kept under greenhouse conditions as controls for monitoring growth dynamics and changes throughout the entire growth period.

After 10 days of low-temperature stress treatment, six pots per resource were randomly selected for physiological parameter measurements. Leaves designated as +1 (containing visible thickened bands, as described by Yang et al., 2020b) with uniform size and phenotype were collected from each material, with three biological replicates per sample. The following parameters were measured: Leaf Relative Water Content (RWC) was determined using the gravimetric method with saturation weighing (Boaretto et al., 2014). Chlorophyll Content (CHL) was measured using an acetone-ethanol extraction method (Yang et al., 2020a). Plasma Membrane Permeability (PMP) was assessed using a conductivity meter (DDSJ-318T, Leici, China). Soluble Protein Content (SP) was quantified using the Coomassie Brilliant Blue G-250 staining method (Ru et al., 2024). Soluble Sugar Content (SS) was determined by the anthrone colorimetric method (Ru et al., 2024). Malondialdehyde (MDA) Content was measured using the thiobarbituric acid (TBA) reaction method, which quantifies MDA by measuring the absorbance of the MDA-TBA complex at 532 nm, with non-specific turbidity corrected by subtracting the absorbance at 600 nm (Wang et al., 2019). Peroxidase (POD) Activity was determined by monitoring the increase in absorbance at 470 nm due to guaiacol oxidation. Superoxide Dismutase (SOD) Activity was evaluated by assessing the inhibition of nitroblue tetrazolium (NBT) photochemical reduction at 560 nm (Wang et al., 2019).

### 2.3 Data analysis

Statistical analyses, including analysis of variance (ANOVA), correlation analysis, normality testing, and cluster analysis, were performed using SPSS 21.0 software. Cluster analysis results were visualized using HemI software, and graphs were generated using Sigmaplot 12. To account for variations in different indices among resources, cold tolerance was evaluated using the cold tolerance coefficient (Yang et al., 2020b), calculated as the ratio of the treatment value to the control value for each index. A comprehensive evaluation of sorghum cold tolerance was conducted using the membership function method from fuzzy mathematics (de Oliveira, 1999), with the following formula:


(1) $$U(X_{ij})=(X_{ij}-X_{jmin})/(X_{jmax}-X_{jmin})$$

(2) $$U(X_{ij})=1-(X_{ij}-X_{jmin})/(X_{jmax}-X_{jmin})$$

(3) $$Di=\frac{\sum_{j}^{\text{n}}=1U(Xij)}{\text{n}}$$

In the formula, *U(X_ij_)* represents the cold tolerance membership function value of the *j*-th index for the *i*-th resource, and *X_ij_* denotes the value of the *j* -th index for the *i* -th resource. *X_jmax_* and *X_jmin_* correspond to the maximum and minimum values of the *j* -th index across all resources, respectively. The mean membership function value, *D_i_*, for the *i* -th resource is calculated as the average of the membership function values across all *n* indices, with membership function values ranging from 0 to 1. For indices positively correlated with cold tolerance,*U(X_ij_)* was computed using Equation 1, whereas Equation 2 was applied for indices negatively correlated with cold tolerance. Finally, *D_i_* was determined as the mean membership function value using Equation 3. A higher *D_i_* value indicates stronger cold tolerance for the corresponding resource.

## 3 Results

### 3.1 Evaluation of cold tolerance based on germination performance

#### 3.1.1 Germination characteristics of sorghum under low temperature

Significant differences were observed among the sorghum resources in terms of germination potential, germination percentage, plumule length, plumule weight, radicle length, and radicle weight (**Supplementary Table 2**). These findings indicate that germination characteristics are closely linked to genetic variability. Furthermore, temperature exerted a profound influence on all measured parameters, with highly significant differences detected across different temperature regimes, underscoring its critical role in shaping germination traits. Notably, germination potential emerged as the most sensitive indicator to temperature stress. At 10°C, all tested resources exhibited a germination potential of zero, whereas the germination percentage showed relatively less sensitivity to temperature variations across the different resources.

To evaluate the impact of different temperatures on sorghum germination traits, we averaged the germination indices of various sorghum resources across three temperature treatments (**Supplementary Table 3**). A decline in germination temperature consistently reduced germination potential, germination percentage, plumule length, radicle length, plumule weight, and radicle weight. Specifically, compared to the control temperature of 25°C, the germination percentage decreased by 45% at 15°C and by 60% at 10°C. Interestingly, the radicle-to-plumule length ratio and radicle-to-plumule weight ratio increased with decreasing temperature, suggesting that plumules are more sensitive to low temperatures than radicles.

#### 3.1.2 Cold tolerance coefficient of sorghum under low temperature

To compare the responses of different sorghum resources to temperature variations, germination indices at 15°C and 10°C were normalized to those at 25°C (control) to calculate cold tolerance coefficients for each trait (**Table 1**). Under low-temperature stress, all measured germination traits exhibited varying degrees of decline across the 71 sorghum resources, with significant differences observed among genotypes. The coefficient of variation ranged from 0.00% to 83.43%, highlighting the rich genetic diversity of the tested materials and the sensitivity of these traits to low temperatures. However, the trends and magnitudes of change in individual traits varied significantly among resources under different temperature treatments. Consequently, evaluating cold tolerance based solely on the cold tolerance coefficient of a single trait is insufficient to accurately represent overall cold tolerance levels or determine optimal temperature conditions for sorghum cold tolerance screening. Therefore, a comprehensive evaluation incorporating multiple traits is necessary for a more reliable assessment.

**Table 1:** Cold tolerance coefficients (CTC) based on germination traits in diverse sorghum germplasms.

CTC by germination trait	Temperature(°C)	Max	Min	Mean	CV(%)
Germination potential	15°C	0.857	0.00	0.289	65.07
	10°C	0.00	0.00	0.00	0.00
Germination percentage	15°C	0.967	0.059	0.560	32.99
	10°C	0.824	0.059	0.403	39.78
Plumule length	15°C	0.485	0.053	0.216	44.64
	10°C	0.410	0.020	0.147	66.80
Radicle length	15°C	0.903	0.107	0.372	44.15
	10°C	0.967	0.099	0.334	50.55
Plumule fresh weight	15°C	0.281	0.024	0.093	55.49
	10°C	0.470	0.002	0.090	83.43
Radicle fresh weight	15°C	0.811	0.063	0.289	52.78
	10°C	0.909	0.023	0.318	59.58

Max, Maximum value; Min, Minimum value; CV, Coefficient of variation.

*CTC, cold tolerance coefficient, calculated as the ratio of the trait value at 10°C or 15°C to that at 25°C. Values are dimensionless.

#### 3.1.3 Membership function analysis of sorghum germination traits under low temperature

The membership function method was employed to integrate multiple indices for classifying and evaluating cold tolerance across different sorghum resources, effectively identifying genotypes with extreme responses to low-temperature stress. Using 25°C as the control, membership function analyses were performed on the cold tolerance coefficients of germination traits for each sorghum resource at 15°C and 10°C (**Supplementary Tables 4**, **5**). The results consistently demonstrated that resources such as 12 and 22 exhibited extremely strong cold tolerance, while resources like 17, 44, and 67 showed the weakest cold tolerance. However, when integrating the membership function values of the 71 sorghum resources across the two temperature treatments, the rankings of cold tolerance were not entirely consistent under different low-temperature conditions, indicating variations in the temperature ranges to which different resources are adapted. Moreover, at excessively low treatment temperatures, most resources, except for a few extreme cases, exhibited minimal differentiation in their responses to cold stress, making it challenging to clearly classify their cold tolerance levels. Therefore, identifying an appropriate temperature condition for evaluating the majority of sorghum resources is crucial for accurate and reliable assessments.

**Figure 2:**
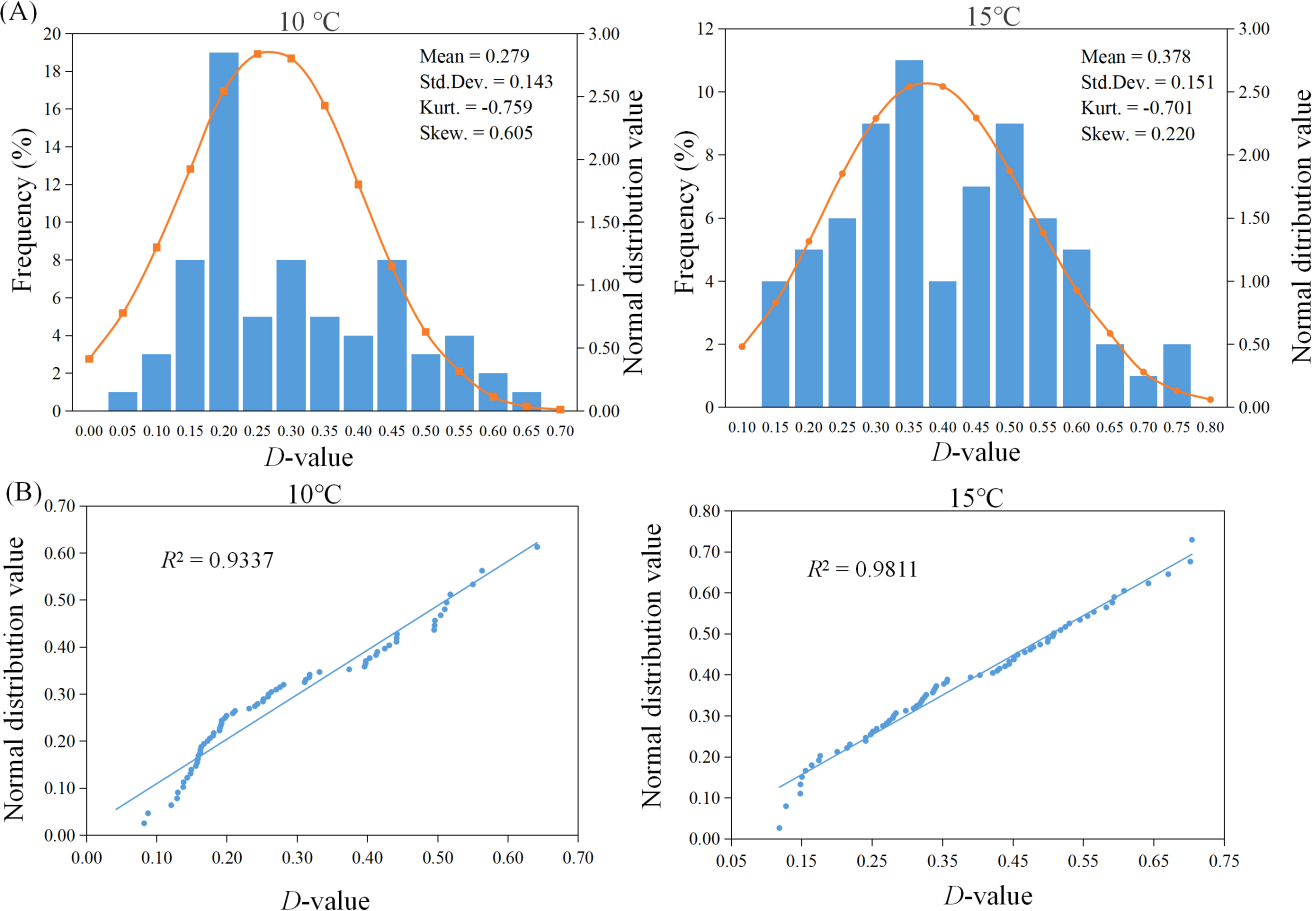
Normal distribution of D-values of 71 sorghum resources after different cold treatments. **(A)** Frequency histogram. **(B)** Quantile-Quantile Plot.

To determine the optimal temperature condition for evaluating cold tolerance during the sorghum germination stage, a statistical analysis was conducted on the comprehensive membership function values of germination performance for 71 sorghum resources under different treatment temperatures. As shown in **Figure 2A**, the peak values of the normal distribution tests for the comprehensive membership functions at both 15°C and 10°C were less than zero. The kurtosis at 15°C was -0.701, with a smaller absolute value compared to the 10°C treatment, indicating a flatter distribution. Additionally, the skewness of the normality tests under both temperature treatments was greater than 1, reflecting significant positive deviations. However, the skewness at 15°C was 0.220, which more closely approximated a normal distribution. The QQ-Plot analysis of membership functions (**Figure 2B**) further confirmed that the membership function values after the 15°C treatment exhibited a high degree of fit to the trend line, with an R² value of 0.9811, closest to 1, indicating compliance with a standard normal distribution. Consequently, 15°C is considered an appropriate low-temperature stress condition for assessing cold tolerance during the sorghum germination stage.

#### 3.1.4 Comprehensive evaluation of cold tolerance during the sorghum germination stage

**Figure 3:**
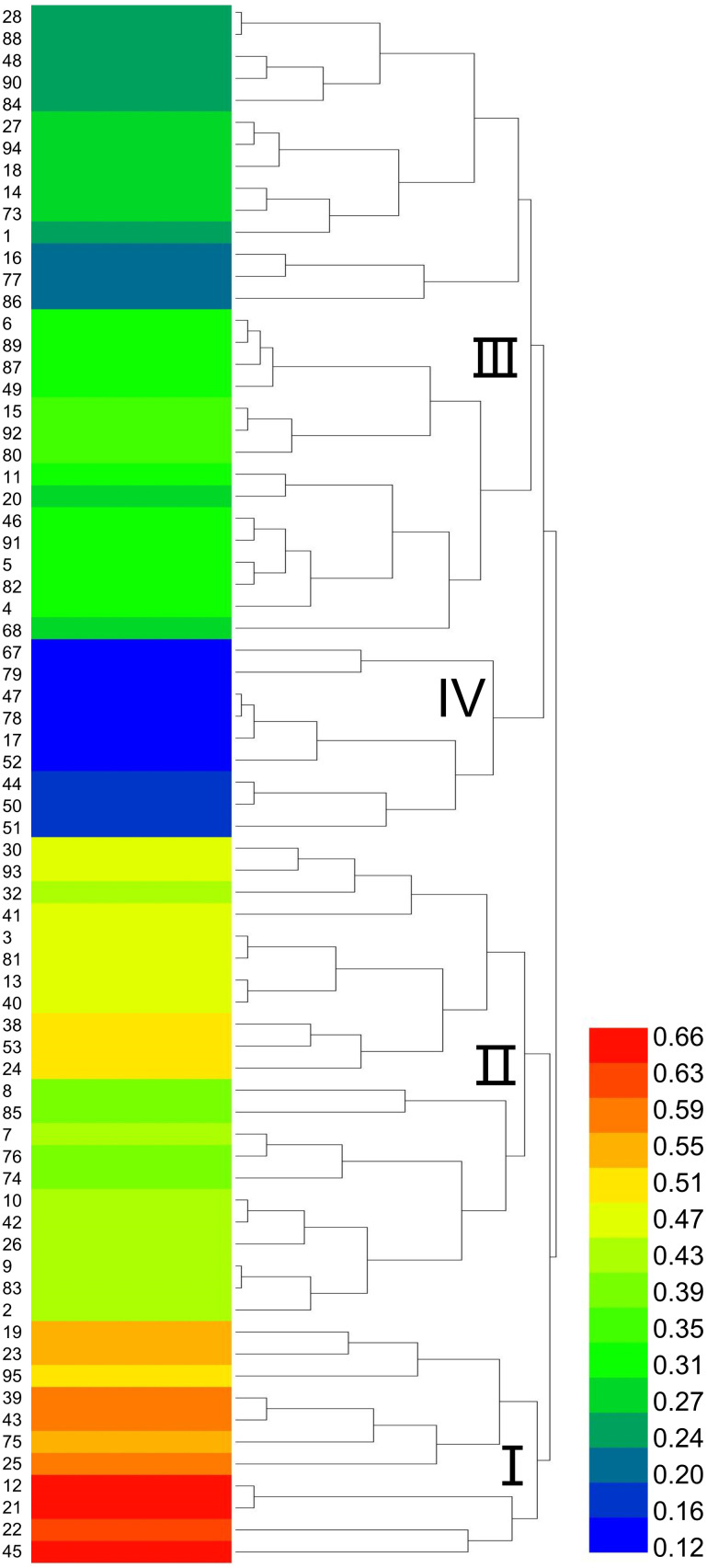
Dendrogram of cold tolerance in 71 sorghum germplasms. I represents the cold-tolerant type. II represents the moderately cold-tolerant type. III represents the moderately sensitive type. IV represents the temperature-sensitive type. The color bar on the right side indicates the range of Z-scores used for the heatmap. The color scale ranges from blue (low Z-score) to red (high Z-score).

Based on the selection of 15°C as the low-temperature stress condition for evaluating cold tolerance in sorghum, a cluster analysis was performed on the comprehensive membership function values of 71 sorghum resources under this treatment (**Figure 3**). The resources were classified into four major groups. Group I, identified as cold-tolerant, included 11 resources with IDs 19, 95, 12, 21, 22, 45, among others. Group II, characterized as moderately cold-tolerant, consisted of 22 resources, including IDs 30, 93, 32, 13, and 2. Group III, classified as moderately cold-sensitive, encompassed 29 resources, such as IDs 28, 88, 48, and 90. Finally, Group IV, identified as cold-sensitive, comprised 9 resources, including IDs 17, 44, 52, and 67.

### 3.2 Evaluation of cold tolerance based on agronomic responses

#### 3.2.1 Variations in field climate during the experiment period

Temperature conditions at the experimental site for 2023 and 2024 are summarized in **Figure 1**. During the seedling emergence stage (February to March), the average temperature ranged from 6.84°C to 14.08°C, with the lowest recorded temperature being -2.90°C and the highest reaching 28.90°C. This period was characterized by a relatively high frequency of low-temperature days, indicating significant temperature variability during this critical phase. In the vegetative growth stage, which spans from jointing stage (6–7 leaf age) to booting stage (12–13 leaf age) (April to June), temperatures steadily increased alongside higher precipitation levels. The average temperature during this period ranged from 18.68°C to 21.95°C, with maximum temperatures reaching up to 33.6°C and minimum temperatures dropping to 7.01°C. By contrast, during the panicle emergence, flowering, and maturation stages (July to August), temperatures continued to rise, averaging around 25°C. Maximum temperatures peaked at 38.2°C, while minimum temperatures were recorded at 15.70°C. These patterns demonstrate that adjusting the sowing date in the experimental region can effectively modulate field-scale temperature exposure. Early sowing reduces temperatures during the early growth stages, whereas delayed sowing increases temperatures, particularly during the seedling stage.

#### 3.2.2 Seedling emergence characteristics of sorghum resources

**Figure 4:**
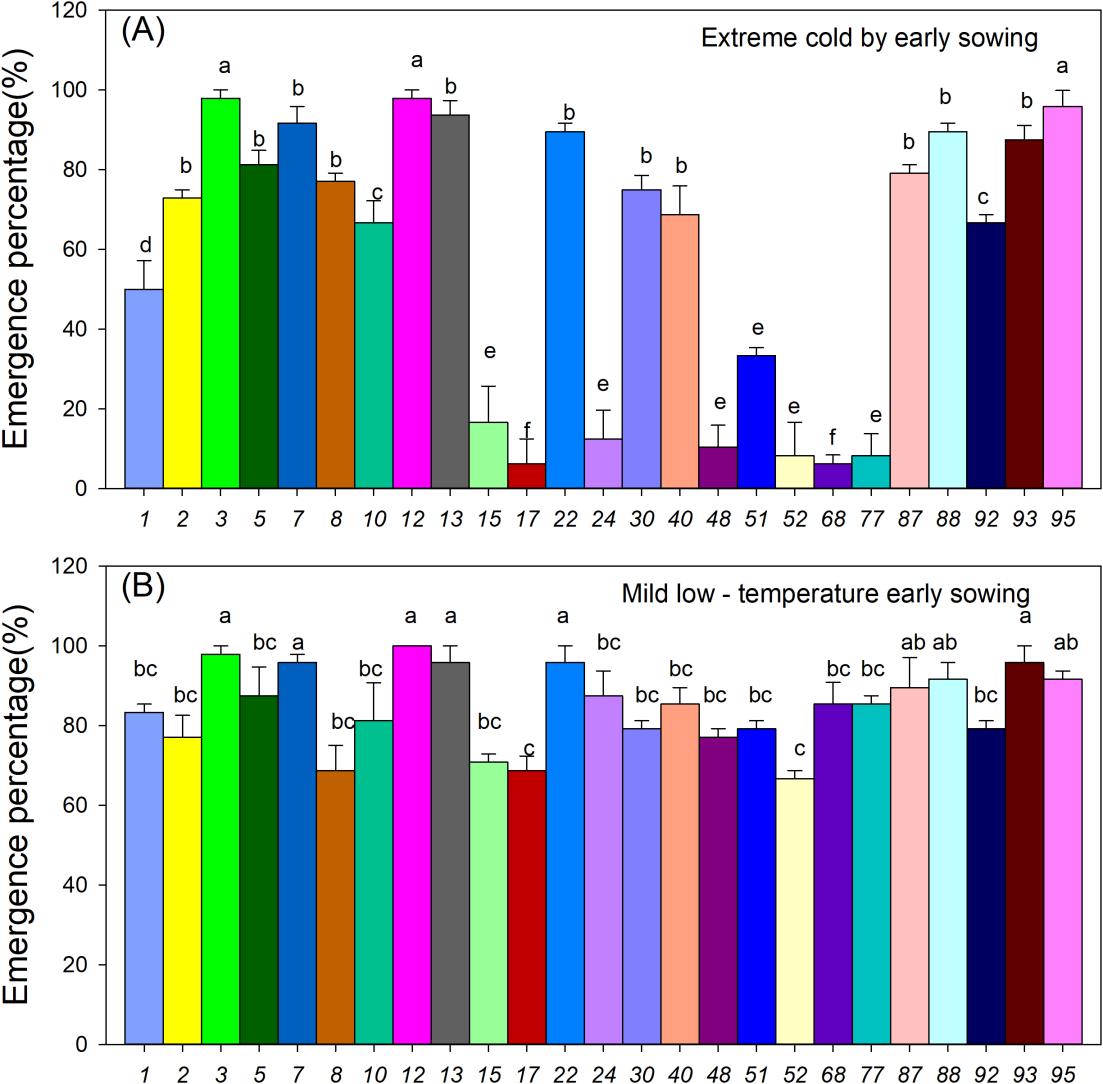
Effect of early sowing on the emergence of 25 sorghum germplasm accessions. **(A)** Seedling emergence rate under extreme cold conditions due to early sowing; **(B)** Seedling emergence rate under mild low-temperature early sowing. Data are the mean values of three replicates. Different letters within each resource row indicate significant differences, as determined by Duncan’s multiple range test (*P* < 0.05).

Early sowing under low-temperature stress significantly affected the seedling emergence of various sorghum germplasm resources (**Figure 4**). Under severe low-temperature conditions with early sowing, only a few resources, such as accessions 3, 12, and 95, exhibited emergence rates exceeding 80%, which were significantly higher than those of accessions 16, 17, 24, 48, 52, 68, and 77, whose emergence rates did not exceed 40% (**Figure 4A**, P < 0.05). In contrast, under mild low-temperature conditions with early sowing, most resources achieved emergence rates around 80%, but the differences in emergence rates among genotypes narrowed (**Figure 4B**). These results indicate that low-temperature stress during early sowing significantly reduces seedling emergence, with significant variation observed among different germplasm materials.

#### 3.2.3 Yield of sorghum resources

Low-temperature stress induced by varying sowing dates significantly reduced the single-panicle grain weight of the evaluated sorghum resources. Significant differences were observed among the germplasm materials, and a significant interaction effect (*P* < 0.05) was detected between sorghum genotypes and sowing dates (**Supplementary Figure 1**). Notably, resources such as 2 (63%, 32%), 12 (38%, 16%), 22 (50%, 23%), 77 (20%, 7%), 88 (48%, 9%), and 93 (49%, 18%) showed substantial increases in single-panicle grain weight under both severe and mild low-temperature early sowing conditions, indicating that these resources exhibit yield improvements under cold stress. In contrast, resources including 7 (10%, 18%), 8 (12%, 36%), 10 (5%, 17%), 15 (19%, 29%), 48 (35%, 14%), 52 (46%, 4%), and 92 (17%, 28%) demonstrated significant declines in grain weight under the same conditions, suggesting their susceptibility to low-temperature stress. These findings highlight the differential responses of sorghum resources to cold stress, emphasizing variability in cold tolerance among genotypes.

#### 3.2.4 Comprehensive evaluation of cold tolerance in sorghum resources under field conditions

**Figure 5:**
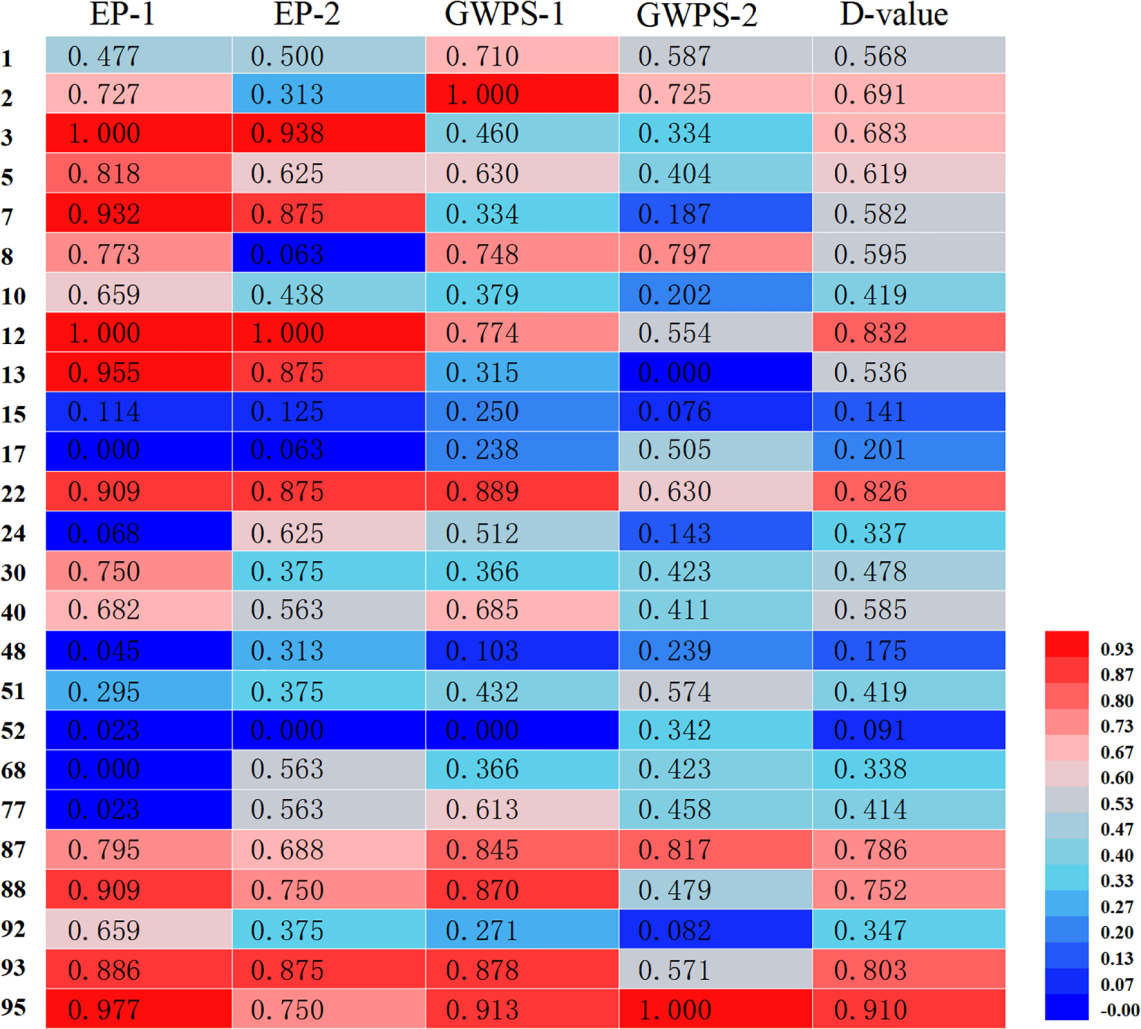
Membership function analysis of field cold tolerance indices in 25 sorghum germplasms. EP-1 and EP-2 represent the cold tolerance coefficients of seedling emergence rates under severe low-temperature early sowing and mild low-temperature early sowing, respectively. GWPS-1 and GWPS-2 represent the cold tolerance coefficients of grain weight per spike under severe and mild low-temperature early sowing conditions, respectively. The D-value represents the mean membership function value for each sorghum germplasm. The color bar on the right side indicates the range of Z-scores used for the heatmap. The color scale ranges from blue (low Z-score) to red (high Z-score).

**Figure 6:**
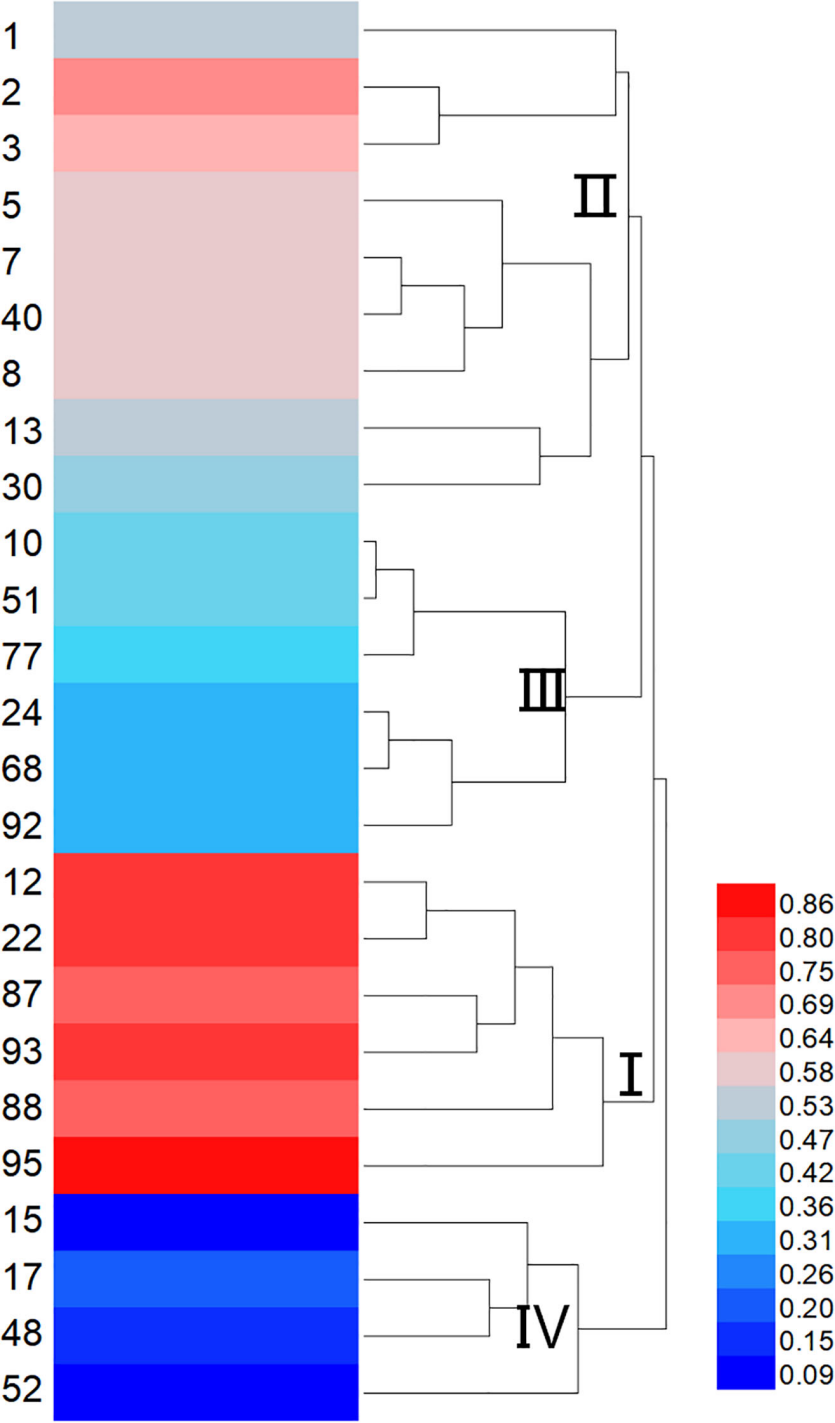
Cluster analysis of field cold tolerance indices in 25 sorghum germplasms. I represents the cold-tolerant type. II represents the moderately cold-tolerant type. III represents the moderately sensitive type. IV represents the temperature-sensitive type. The color bar on the right side indicates the range of Z-scores used for the heatmap. The color scale ranges from blue (low Z-score) to red (high Z-score).

Membership function analysis was conducted on the cold tolerance coefficients of seedling emergence rate and single-panicle grain weight for each sorghum resource under severe and mild low-temperature early sowing conditions, using conventional sowing as the control (**Figure 5**). The comprehensive membership function values (D-values) for resources 2, 3, 5, 12, 22, 87, 88, 93, and 95 were all above 0.6, indicating strong cold tolerance under field conditions. Conversely, resources 15, 17, 48, and 52 exhibited lower average D-values, suggesting higher sensitivity to low-temperature stress. A cluster analysis of the comprehensive membership function values for the 25 sorghum resources (**Figure 6**) classified them into four major groups: Group I, identified as cold-tolerant, included six resources (e.g., 12, 22, and 95); Group II, moderately cold-tolerant, comprised nine resources (e.g., 1, 2, and 30); Group III, moderately cold-sensitive, included six resources (e.g., 10, 51, and 92); and Group IV, cold-sensitive, consisted of four resources (e.g., 15 and 17). This classification highlights the variability in cold tolerance among the evaluated sorghum resources.

**Figure 7:**
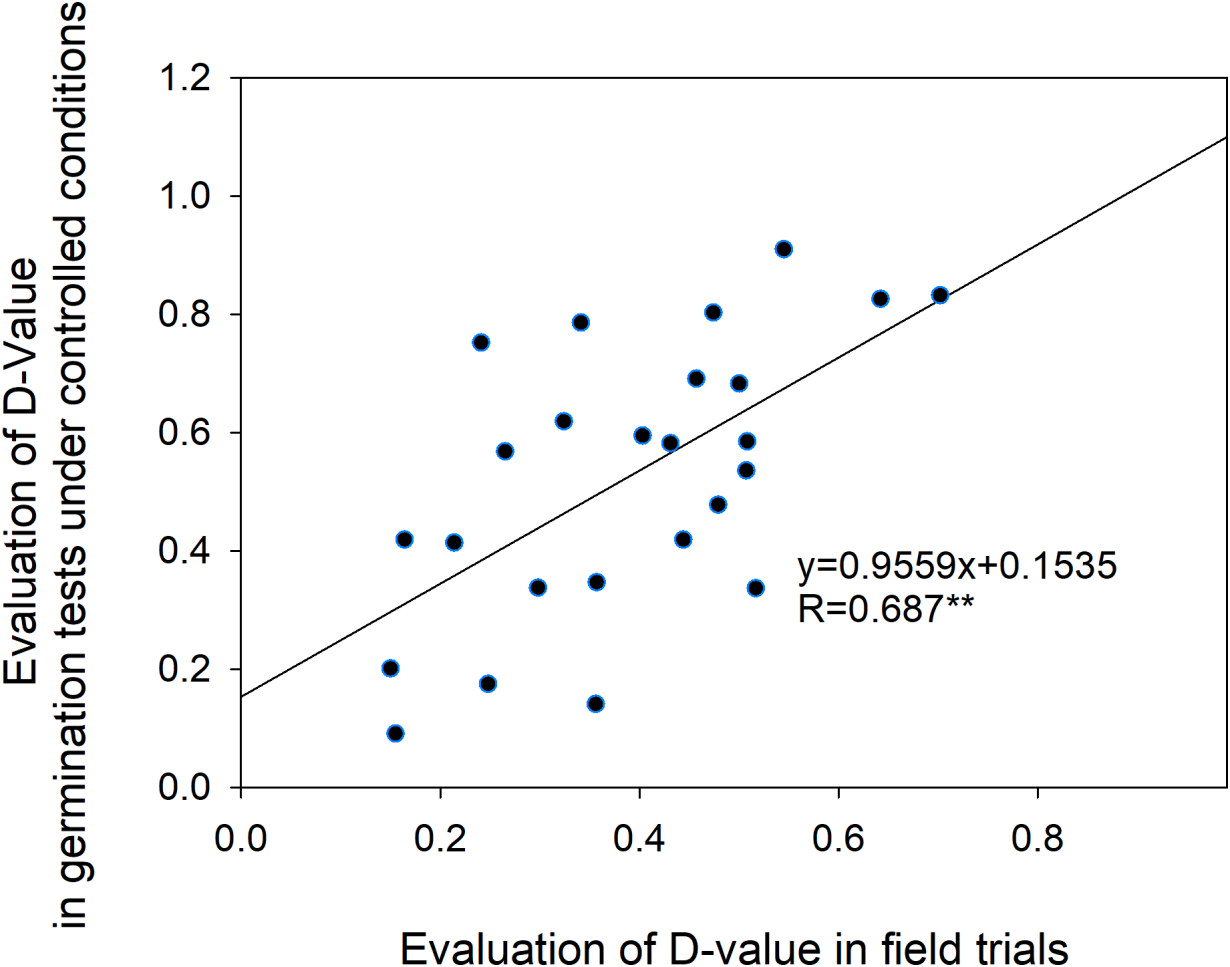
Correlation between indoor seed germination cold tolerance and field-based cold tolerance.

To further validate the consistency between cold tolerance evaluations based on agronomic responses and germination performance, a correlation analysis was performed. The results revealed a significant positive correlation (*r* = 0.687, *P* < 0.05) between cold tolerance assessed through seed germination under controlled indoor conditions and field-based agronomic cold tolerance (**Figure 7**). This indicates that evaluation outcomes from both approaches are consistent, suggesting that indoor germination-based cold tolerance assessments can serve as a reliable proxy for field-based agronomic cold tolerance evaluations under certain conditions.

### 3.3 Physiological responses and cold tolerance evaluation of sorghum germplasm resources under low-temperature stress

#### 3.3.1 Physiological responses of different sorghum resources to low-temperature stress

To further evaluate the cold tolerance of sorghum resources selected during the germination stage and classified as cold-tolerant, intermediate, and cold-sensitive types, a growth chamber experiment was conducted at the seedling stage using physiological indices. Following low-temperature stress, relative water content (RWC) and chlorophyll content (CHL) decreased, while relative electrolyte leakage (PMP), superoxide dismutase (SOD), peroxidase (POD), malondialdehyde (MDA), soluble protein (SP), and soluble sugars (SS) increased. Significant differences were observed in all physiological parameters between the low-temperature treatment and the control (**Supplementary Table 6**). These results indicate that low-temperature stress significantly affects the physiological traits of different sorghum resources, though the extent of these effects varies, highlighting significant differences in cold tolerance among the evaluated genotypes.

To quantify the differential responses of various sorghum resources to low-temperature stress based on physiological traits, cold tolerance coefficients for each physiological index were calculated under low-temperature stress (10°C) relative to a control condition (25°C) (**Table 2**). After exposure to low-temperature stress, RWC and CHL showed reductions compared to pre-stress levels (cold tolerance coefficient < 1), whereas PMP, SS, SP, POD, and SOD exhibited increases (cold tolerance coefficient > 1). The variation in the maximum and minimum values of the cold tolerance coefficients across the eight physiological indices reflected diverse responses among the evaluated sorghum resources.

**Table 2:** Comparison of cold tolerance coefficients for various physiological indicators.

Material	RWC	PMP	CHL	SOD	POD	MDA	SP	SS
1	0.946 e	1.161 b	0.926 b	1.504 c	1.365 de	1.342 cd	1.132 a	1.259 d
2	0.980 abc	1.084 b	0.939 b	1.682 ab	1.835 b	1.296 cd	1.052 c	1.289 d
3	0.963 d	1.123 b	0.945 b	1.547 bc	1.525 cd	1.414 c	1.038 c	2.437 c
12	0.986 a	1.095 b	0.976 a	1.766 a	2.026 a	1.202 cd	1.118 ab	3.354 a
13	0.970 cd	1.170 b	0.936 b	1.523 bc	1.691 bc	1.249 cd	1.059 c	1.428 d
15	0.966 d	1.191 b	0.918 b	1.298 d	1.293 ef	1.797 a	1.033 c	1.259 d
17	0.966 d	1.297 a	0.912 b	1.286 d	1.151 f	1.644 b	1.028 c	1.103 d
22	0.972 bcd	1.094 b	0.934 b	1.658 abc	1.796 b	1.173 d	1.034 c	2.735 b
95	0.984 ab	1.150 b	0.910 b	1.641 abc	1.510 cd	1.384 cd	1.084 bc	1.390 d
Mean	0.970	1.152	0.933	1.545	1.577	1.389	1.064	1.806
CV (%)	1.336	6.547	2.523	10.865	18.008	15.158	3.862	44.075
P-value	***	**	***	*	ns	*	**	ns

RWC, leaf relative water conent; PMP, plasma membrane permeability; CHL, chlorophyll content; SOD, catalase; POD, peroxidase; MDA, malondialdehyde; SP, soluble protein; SS, soluble sugar. Means followed by different lowercase letters within the same row are significantly different (*P* < 0.05) according to the SNK test. *P < 0.05, **P < 0.01, ***P < 0.001, ns, not significant (*P* > 0.05).

Correlation analysis of the cold tolerance coefficients for each physiological index (**Supplementary Table 7**) revealed a significant positive correlation between the cold tolerance coefficients of relative electrolyte leakage (PMP) and malondialdehyde (MDA), with a correlation coefficient of 0.523 (*P <*0.01). However, these two indices were negatively correlated with the cold tolerance coefficients of the other six physiological indices. This suggests that PMP and MDA are closely related indicators of membrane damage under cold stress, while other indices such as RWC, CHL, and antioxidant enzyme activities provide complementary information on the mechanisms underlying cold tolerance in sorghum.

#### 3.3.2 Comprehensive evaluation of cold tolerance in sorghum resources based on physiological responses

Membership function analysis was conducted on the cold tolerance coefficients of each physiological index. For indices negatively correlated with cold tolerance, such as relative electrolyte leakage (PMP) and malondialdehyde (MDA), fuzzy membership function values were calculated using Equation 2. Conversely, for indices positively correlated with cold tolerance, such as relative water content (RWC), chlorophyll content (CHL), superoxide dismutase (SOD), peroxidase (POD), soluble protein (SP), and soluble sugars (SS), fuzzy membership function values were calculated using Equation 1. The results (**Table 3**) indicate that cold tolerance among the evaluated resources was ranked based on their D-values. Accession 17 exhibited the lowest D-value of 0.097, marking it as the least cold-tolerant resource. In contrast, accession 12 had the highest D-value of 0.971, indicating it as the most cold-tolerant.

**Table 3:** Membership function analysis of different sorghum germplasms based on physiological cold tolerance indices.

Material	RWC	PMP	CHL	SOD	POD	MDA	SP	SS	D-value	Ranking
1	0.000	0.639	0.239	0.455	0.245	0.729	1.003	0.069	0.422	6
2	0.852	1.002	0.438	0.826	0.782	0.802	0.228	0.082	0.627	3
3	0.417	0.818	0.529	0.543	0.428	0.614	0.095	0.592	0.505	5
12	1.002	0.946	1.008	1.000	1.000	0.953	0.862	1.000	0.971	1
13	0.604	0.596	0.394	0.494	0.617	0.879	0.295	0.144	0.503	5
15	0.490	0.496	0.128	0.026	0.163	0.001	0.045	0.069	0.177	7
17	0.494	0.000	0.033	0.001	0.000	0.245	0.002	0.000	0.097	8
22	0.653	0.952	0.358	0.774	0.737	0.999	0.057	0.725	0.657	2
95	0.959	0.690	0.005	0.739	0.411	0.663	0.542	0.127	0.517	4

RWC, leaf relative water conent; PMP, plasma membrane permeability; CHL, chlorophyll content; SOD, catalase; POD, peroxidase; MDA, malondialdehyde; SP, soluble protein; SS, soluble sugar. D-value, Average of fuzzy membership values.

Hierarchical cluster analysis using the Euclidean distance method based on D-values classified the nine sorghum resources into four groups (**Supplementary Figure 2**). One resource, accession 12, clustered into Group I, representing the highly cold-tolerant type. Six resources-accessions 1, 2, 3, 13, 22, and 95-were grouped into Cluster II, representing the cold-tolerant type. The remaining two resources, accessions 15 and 17, were grouped into Cluster III, representing the cold-sensitive type.

#### 3.3.3 Correlation analysis between physiological responses and cold tolerance

**Figure 8:**
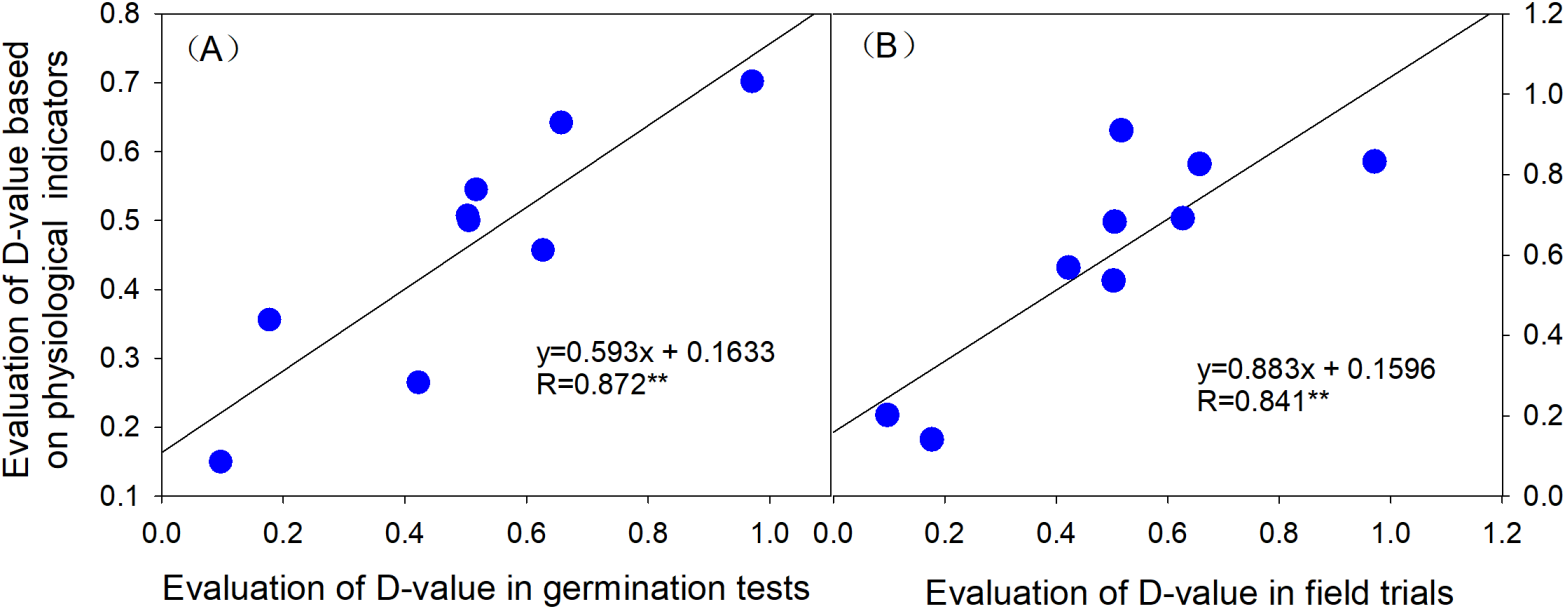
Correlation analysis of cold tolerance based on physiological indices with indoor seed germination cold tolerance and field cold tolerance. **(A)** Correlation between cold tolerance based on physiological indices and indoor seed germination cold tolerance. **(B)** Correlation between cold tolerance based on physiological indices and field cold tolerance.

To further validate the consistency between cold tolerance evaluations based on physiological indices, germination performance, and field trial results, a correlation analysis was conducted between the physiological index-based cold tolerance evaluation and the comprehensive assessments of cold tolerance derived from seed germination (indoor) and field trials. The results (**Figure 8**) demonstrate that the cold tolerance evaluation values based on physiological indices were positively correlated with both indoor seed germination cold tolerance and field-based cold tolerance evaluations, with correlation coefficients exceeding 0.8 (*P* < 0.01). Notably, the correlation between physiological indices and indoor seed germination cold tolerance was slightly higher, reaching 0.872 (*P* < 0.01).

To identify robust physiological indicators for cold tolerance evaluation and to explore the potential for establishing a mathematical model, stepwise regression analysis was performed. The comprehensive cold tolerance evaluation D-values from field trials served as the dependent variable, while the cold tolerance coefficients of various physiological indices acted as independent variables. Stepwise regression identified the optimal regression equation as: D = -2.261 + 2.459SOD - 0.596POD (*R*²= 0.954, *P* < 0.001)., with an R² value of 0.954 (*P* < 0.001). Among the eight examined physiological indices-POD, SS, SP, PMP, etc.-only superoxide dismutase (SOD) and peroxidase (POD) significantly influenced the cold tolerance of different sorghum resources.

**Figure 9:**
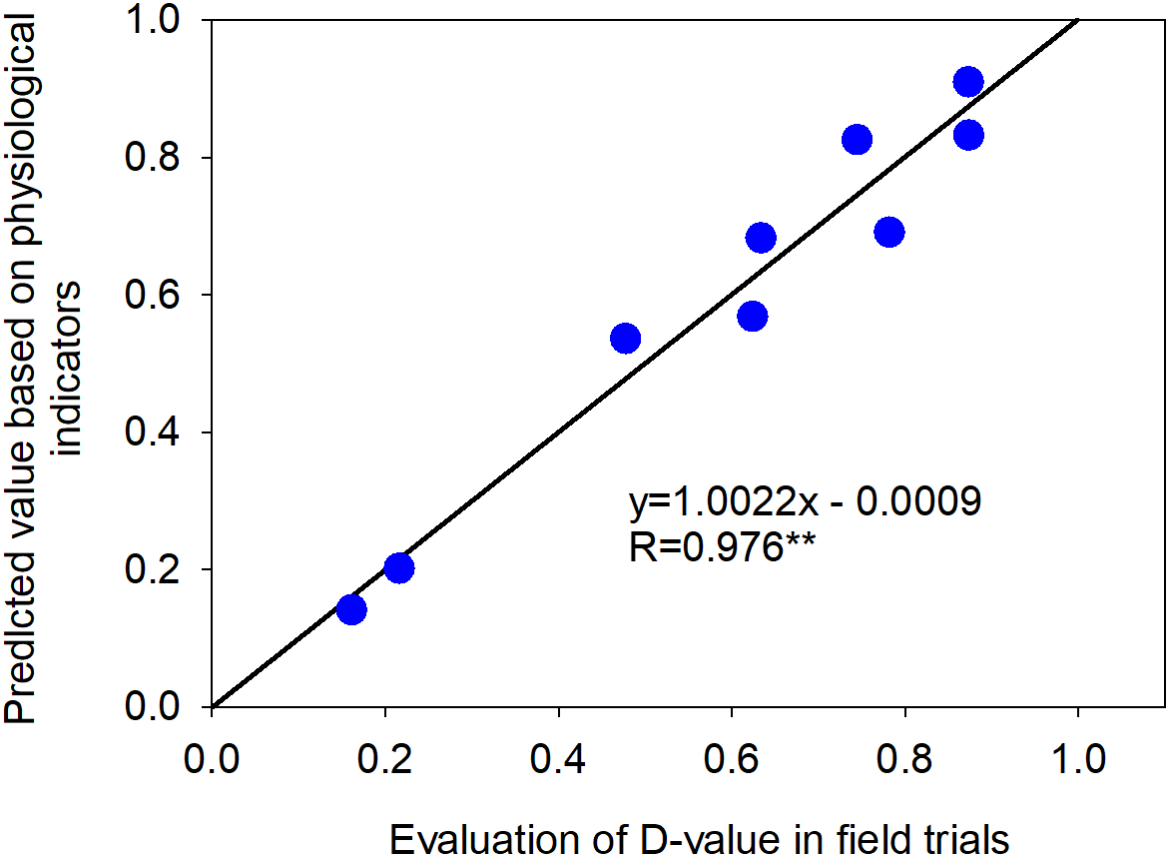
Correlation between the D-value of field cold tolerance and the predicted D-value of cold tolerance based on physiological indices.

The physiological index values of nine liquor-use sorghum resources were substituted into the stepwise regression equation to predict their cold tolerance D-values. Subsequently, the correlation between field-based cold tolerance D-values and the predicted D-values was analyzed. The results showed a highly significant correlation (*P* < 0.01) with an r-value of 0.976 (**Figure 9**), indicating that the stepwise regression equation based on physiological indices can effectively evaluate the cold tolerance of different liquor-use sorghum resources.

## 4 Discussion

Low-temperature stress represents a critical abiotic challenge that significantly impacts crop productivity worldwide. Recently efforts to identify cold-tolerant sorghum germplasm have gained traction in various regions globally (Fiedler et al., 2014; Parra-Londono et al., 2018; Vera Hernández et al., 2023; Neitzert et al., 2025). However, this area of research has received relatively limited attention within China. Sorghum cultivation in China is predominantly for brewing purposes, especially in the Chishui River Basin of the southwestern region, an area renowned for its abundant local sorghum germplasm resources (Yan et al., 2018) and celebrated for producing world-famous sauce-flavored liquor (Shi et al., 2024; Zhou et al., 2020). Despite the economic importance of this region, the cold tolerance of its sorghum resources remains poorly understood. The burgeoning demand for sauce-flavored liquor necessitates expanding sorghum cultivation into cooler, high-altitude areas beyond the Chishui River Basin. Moreover, early-season sorghum growth in the basin is frequently jeopardized by late spring cold snaps. These challenges underscore the necessity for developing cold-tolerant sorghum varieties and enhancing low-temperature stress management practices. In our study, we systematically evaluated the cold tolerance of 71 sorghum germplasm resources primarily from the Chishui River Basin, focusing on germination performance and field agronomic responses. The results revealed substantial variation in cold tolerance among the evaluated genotypes. Notably, cold-tolerant materials such as accessions 12 and 22 demonstrated robust adaptability across germination traits, field agronomic performance, and physiological responses under low-temperature stress. Conversely, cold-sensitive genotypes, including No.17, 44, and 48, exhibited significant reductions in germination percentage, seedling emergence, and yield under similar conditions. These findings highlight the potential of local germplasm resources from the Chishui River Basin to enhance cold tolerance and resistance in sorghum. Our work provides valuable insights for breeding programs aimed at developing cold-tolerant sorghum varieties specifically tailored for liquor production in southwestern China. Furthermore, it offers guidance for optimizing regional cultivation layouts and refining low-temperature stress management strategies.

The question arises as to why the Chishui River Basin harbors such a rich repository of cold-tolerant sorghum resources. This phenomenon is likely closely tied to the region’s distinctive topographical and climatic conditions. Situated on the Yunnan-Guizhou Plateau in southwestern China, the basin spans an elevation range of 300–1,700 meters, where late spring cold snaps are common. These environmental conditions create a natural selection pressure that favors the development of cold-tolerant sorghum germplasm (Maulana et al., 2017). Additionally, the area’s long-standing tradition of liquor production has been accompanied by extensive cultivation of sorghum over extended periods, fostering a genetically diverse population (Li et al., 2025). Notably, most sorghum varieties grown in this region are glutinous types, characterized by high levels of amylopectin and a higher proportion of amylopectin relative to total starch. Evidence from other crops suggests that cold environments often favor the prevalence of strongly glutinous varieties. For instance, rice cultivated in high-altitude, cool regions tends to exhibit stronger glutinous properties (Xu et al., 2020; Zhu et al., 2017). This implies that the unique climatic conditions of the Chishui River Basin may have contributed to the enrichment of glutinous sorghum varieties with enhanced cold tolerance. Exploring cold-tolerant germplasm within these glutinous sorghum varieties used for brewing could provide valuable insights for breeding cold-tolerant sorghum intended for other purposes, such as feed or food. Importantly, this also underscores the need to investigate the relationship between cold tolerance and brewing quality in sorghum germplasm. Utilizing cold-tolerant sorghum resources could potentially enhance both the resilience and brewing performance of sorghum, offering significant implications for future breeding programs.

A reliable method for evaluating cold tolerance in crops is crucial for identifying and utilizing cold-tolerant sorghum germplasm resources. Previous studies have predominantly focused on assessing sorghum seedling germination and growth under controlled low-temperature conditions (Bekele et al., 2014). However, these studies often lack standardized evaluation parameters, relying instead on association analyses to explore potential genetic mechanisms underlying cold tolerance. Moreover, few studies have examined the responses of different sorghum genotypes to low temperatures under field conditions. Elango et al. (2022) and Parra-Londono et al. (2018) have investigated the phenotypic performance of sorghum under early cold stress in field conditions, but these studies mainly focus on seedling emergence and survival, did not incorporate agronomic traits, particularly yield-related traits, which are critical for evaluating cold tolerance under field conditions. Although seedling traits are indicative of comprehensive yield performance, relying solely on these traits under low-temperature stress is insufficient for a holistic assessment of cold tolerance in sorghum (Casto et al., 2021). In this study, we adopted a multi-level approach by first conducting germination experiments under controlled low-temperature conditions in a growth chamber and then simulating low-temperature stress in the field by advancing the sowing date (**Figure 1**). Cold tolerance was evaluated based on agronomic traits such as yield, plant height, and biomass, using evaluation systems constructed from cold tolerance coefficients and membership functions. The correlation between results from germination traits and field-based agronomic traits validated the reliability of our evaluation methods. This comprehensive approach ensures robust and consistent assessments of cold tolerance. Importantly, incorporating agronomic responses into the evaluation provides practical insights for breeding new varieties and optimizing crop management strategies from a field production perspective. Our correlation analysis revealed a significant relationship between cold tolerance evaluations based on seedling germination performance and agronomic traits (**Table 1**), providing additional evidence for leveraging phenotypic correlations, marker-assisted selection, and precise phenotyping to enhance cold tolerance in sorghum breeding programs (Bekele et al., 2014; La Borde et al., 2023; Vera Hernández et al., 2023).

Unlike previous studies, this research not only integrated agronomic responses under field conditions but also explored the physiological responses of sorghum germplasm to low-temperature stress. This dual focus sheds light on the physiological adaptation mechanisms of sorghum to cold stress and further validates the results of cold tolerance evaluations based on germination traits and agronomic responses. Physiological response data indicated that cold-tolerant sorghum genotypes exhibit stable membrane permeability, maintain chlorophyll content, synthesize soluble substances, and enhance antioxidant capacity under low-temperature stress (**Table 2**), thereby mitigating cold-induced damage (Bekele et al., 2014; Ghosh et al., 2024). Distinct from prior studies, this research concentrated on local sorghum resources from the Chishui River Basin, most of which are used for brewing sauce-flavored liquor. Sauce-flavored liquor is renowned worldwide for its rich flavor profile, primarily originating from secondary metabolites in sorghum, including tannins, phenolic compounds, and flavonoids (Kimani et al., 2020; Shi et al., 2024; Wang et al., 2016). Given that the accumulation of tannins, phenolic compounds, and flavonoids is often associated with the physiological processes of crop stress adaptation (Khare et al., 2020; Tharayil et al., 2011), and evidence has shown that cold stress induced by early sowing can significantly improve the physicochemical properties related to brewing quality in sorghum grain (Yang et al., 2025), identifying cold-tolerant sorghum germplasm may simultaneously enhance the quality of sorghum used for liquor production.

## 5 Conclusion

This study comprehensively evaluated cold tolerance in 71 sorghum germplasm accessions, primarily from the Chishui River Basin, using integrated assessments of seed germination, field agronomic performance, and physiological traits under low-temperature stress. Significant variation in cold tolerance was observed, with accessions 12 and 22 exemplifying strong tolerance, exhibiting robust germination and seedling vigor (even at 15 °C) and minimal yield reductions (16-38%) under early sowing/cold conditions, while sensitive accessions (e.g., 17 and 44) showed severe germination inhibition at 10 °C and >50% yield loss. The strong correlation between laboratory germination assays and field performance (R=0.687, *P* < 0.05) validates indoor screening, with 15 °C identified as an optimal germination-stage evaluation temperature. Physiologically, cold tolerance was linked to enhanced oxidative stress regulation, particularly via elevated SOD and POD activities. These findings provide valuable cold-tolerant germplasm (e.g., accessions 12, 22) and reliable screening methods for sorghum breeding, with implications for optimizing cultivation and stress management in southwestern China, especially for liquor-production sorghum. Given the Chishui River Basin’s significance for sauce-flavored liquor production, we recommend further research to explore associations between cold-tolerant germplasm and brewing quality traits, to better align breeding goals with industrial needs.

## Data Availability

The original contributions presented in the study are included in the article/**Supplementary Material**. Further inquiries can be directed to the corresponding authors.
