# Supplementary material for: Evaluation of cold tolerance in sorghum germplasm from the Chishui River Basin in China: insights from germination, field trials, and physiological assays
Source: Front Plant Sci. 2025 Sep 2;16:1630271. doi: 10.3389/fpls.2025.1630271 (PMC12436481; doi:10.3389/fpls.2025.1630271)
Supplement: Supplementary file 1 [file Table1.doc]

Supplementary Table 1. The number and origin of Sorghum variety resources.

| No.of material | Genotype | Origin |
| --- | --- | --- |
| 1 | **Hongyingzi Genetic Variant** | **Daba Town, Renhuai City, Zunyi City, Guizhou Province, China** |
| 2 | **Hongyingzi Genetic Variant** | **Daba Town, Renhuai City, Zunyi City, Guizhou Province, China** |
| 3 | **Hongyingzi Genetic Variant** | **Daba Town, Renhuai City, Zunyi City, Guizhou Province, China** |
| 4 | **Hongyingzi Genetic Variant** | **Changgang Town, Renhuai City, Zunyi City, Guizhou Province, China** |
| 5 | **Hongyingzi Genetic Variant** | **Changgang Town, Renhuai City, Zunyi City, Guizhou Province, China** |
| 6 | **Hongyingzi Genetic Variant** | **Changgang Town, Renhuai City, Zunyi City, Guizhou Province, China** |
| 7 | **Hongyingzi Genetic Variant** | **Erlang Town, Xishui County, Guizhou Province, China** |
| 8 | **Hongyingzi Genetic Variant** | **Erlang Town, Xishui County, Guizhou Province, China** |
| 9 | **Hongyingzi Genetic Variant** | **Erlang Town, Xishui County, Guizhou Province, China** |
| 10 | **Hongyingzi Genetic Variant** | **Erlang Town, Xishui County, Guizhou Province, China** |
| 11 | **Hongyingzi Genetic Variant** | **Luban Town, Renhuai City, Zunyi City, Guizhou Province, China** |
| 12 | **Hongyingzi Genetic Variant** | **Datian Township, Jinsha County, Bijie City, Guizhou Province, China** |
| 13 | **Hongyingzi Genetic Variant** | **Luban Town, Renhuai City, Zunyi City, Guizhou Province, China** |
| 14 | **Hongyingzi Genetic Variant** | **Datian Township, Jinsha County, Bijie City, Guizhou Province, China** |
| 15 | **Hongyingzi Genetic Variant** | **Luban Town, Renhuai City, Zunyi City, Guizhou Province, China** |
| 16 | **Hongyingzi Genetic Variant** | **Datian Township, Jinsha County, Bijie City, Guizhou Province, China** |
| 17 | **Hongyingzi Genetic Variant** | **Luban Town, Renhuai City, Zunyi City, Guizhou Province, China** |
| 18 | **Hongyingzi Genetic Variant** | **Chengguan Town, Jinsha County, Bijie City, Guizhou Province, China** |
| 19 | **Hongyingzi Genetic Variant** | **Luban Town, Renhuai City, Zunyi City, Guizhou Province, China** |
| 20 | **Hongyingzi Genetic Variant** | **Luban Town, Renhuai City, Zunyi City, Guizhou Province, China** |
| 21 | **Hongyingzi Genetic Variant** | **Chengguan Town, Jinsha County, Bijie City, Guizhou Province, China** |
| 22 | **Locally Cultivated Common Sorghum** | **Yingwuxi Town, Sinan County, Tongren City, Guizhou Province, China** |
| 23 | **Hongyingzi Genetic Variant** | **Wuma Town, Renhuai City, Zunyi City, Guizhou Province, China** |
| 24 | **Locally Cultivated Glutinous Sorghum** | **Yingwuxi Town, Sinan County, Tongren City, Guizhou Province, China** |
| 25 | **Hongyingzi Genetic Variant** | **Chengguan Town, Jinsha County, Bijie City, Guizhou Province, China** |
| 26 | **Locally Cultivated Glutinous Sorghum** | **Sinan County, Guizhou Province, China** |
| 27 | **Hongyingzi Genetic Variant** | **Luban Town, Renhuai City, Zunyi City, Guizhou Province, China** |
| 28 | **Hongyingzi Genetic Variant** | **Luban Town, Renhuai City, Zunyi City, Guizhou Province, China** |
| 30 | **Hongyingzi Genetic Variant** | **Changgang Town, Renhuai City, Zunyi City, Guizhou Province, China** |
| 32 | **Hongyingzi Sorghum** | **Dazhi Seed Industry Co., Ltd., Jinzhong City, Shanxi Province, China** |
| 38 | **Hongyingzi Genetic Variant** | **Luban Town, Renhuai City, Zunyi City, Guizhou Province, China** |
| 39 | **Hongyingzi Genetic Variant** | **Luban Town, Renhuai City, Zunyi City, Guizhou Province, China** |
| 40 | **Locally Cultivated Glutinous Sorghum** | **Dejiang County, Tongren City, Guizhou Province, China** |
| 41 | **Hongyingzi Genetic Variant** | **Wuma Town, Renhuai City, Zunyi City, Guizhou Province, China** |
| 42 | **Locally Cultivated Common Sorghum** | **Dejiang County, Tongren City, Guizhou Province, China** |
| 43 | **Hongyingzi Genetic Variant** | **Wuma Town, Renhuai City, Zunyi City, Guizhou Province, China** |
| 44 | **Hongyingzi Genetic Variant** | **Wuma Town, Renhuai City, Zunyi City, Guizhou Province, China** |
| 45 | **Hongyingzi Genetic Variant** | **Wuma Town, Renhuai City, Zunyi City, Guizhou Province, China** |
| 46 | **Hongyingzi Genetic Variant** | **Wuma Town, Renhuai City, Zunyi City, Guizhou Province, China** |
| 47 | **Hongyingzi Genetic Variant** | **Wuma Town, Renhuai City, Zunyi City, Guizhou Province, China** |
| 48 | **Locally Cultivated Broom Sorghum** | **Dejiang County, Tongren City, Guizhou Province, China** |
| 49 | **Locally Cultivated Common Sorghum** | **Dejiang County, Tongren City, Guizhou Province, China** |
| 50 | **Locally Cultivated Common Sorghum** | **Dejiang County, Tongren City, Guizhou Province, China** |
| 51 | **Locally Cultivated Common Sorghum** | **Dejiang County, Tongren City, Guizhou Province, China** |
| 52 | **Locally Cultivated Common Sorghum** | **Dejiang County, Tongren City, Guizhou Province, China** |
| 53 | **Locally Cultivated Broom Sorghum** | **Dejiang County, Tongren City, Guizhou Province, China** |
| 67 | **Hongmao 6** | **Hebei Hanqing Seed Industry Technology Co., Ltd., Qing County, Cangzhou City, Hebei Province, China** |
| 68 | **Hongyingzi Sorghum** | **Hebei Hanqing Seed Industry Technology Co., Ltd., Qing County, Cangzhou City, Hebei Province, China** |
| 73 | **Hongyingzi Sorghum** | **Xingnong Seedlings Co., Ltd., Botou City, Cangzhou City, Hebei Province, China** |
| 74 | **Hongyingzi Genetic Variant** | **Xuekong Town, Renhuai City, Guizhou Province, China** |
| 75 | **Hongyingzi Genetic Variant** | **Xuekong Town, Renhuai City, Guizhou Province, China** |
| 76 | **Hongyingzi Genetic Variant** | **Xuekong Town, Renhuai City, Guizhou Province, China** |
| 77 | **Hongyingzi Genetic Variant** | **Xuekong Town, Renhuai City, Guizhou Province, China** |
| 78 | **Hongyingzi Genetic Variant** | **Luban Town, Renhuai City, Zunyi City, Guizhou Province, China** |
| 79 | **Hongyingzi Genetic Variant** | **Xuekong Town, Renhuai City, Guizhou Province, China** |
| 80 | **Hongyingzi Genetic Variant** | **Luban Town, Renhuai City, Zunyi City, Guizhou Province, China** |
| 81 | **Hongyingzi Genetic Variant** | **Luban Town, Renhuai City, Zunyi City, Guizhou Province, China** |
| 82 | **Hongyingzi Genetic Variant** | **Luban Town, Renhuai City, Zunyi City, Guizhou Province, China** |
| 83 | **Hongyingzi Genetic Variant** | **Luban Town, Renhuai City, Zunyi City, Guizhou Province, China** |
| 84 | **Hongyingzi Genetic Variant** | **Tongzi County, Zunyi City, Guizhou Province, China** |
| 85 | **Hongyingzi Genetic Variant** | **Tongzi County, Zunyi City, Guizhou Province, China** |
| 86 | **Hongyingzi Genetic Variant** | **Tongzi County, Zunyi City, Guizhou Province, China** |
| 87 | **Hongyingzi Genetic Variant** | **Tongzi County, Zunyi City, Guizhou Province, China** |
| 88 | **Hongyingzi Genetic Variant** | **Tongzi County, Zunyi City, Guizhou Province, China** |
| 89 | **Hongyingzi Genetic Variant** | **Tongzi County, Zunyi City, Guizhou Province, China** |
| 90 | **Hongyingzi Genetic Variant** | **Tongzi County, Zunyi City, Guizhou Province, China** |
| 91 | **Hongyingzi Genetic Variant** | **Luban Town, Renhuai City, Zunyi City, Guizhou Province, China** |
| 92 | **Hongyingzi Genetic Variant** | **Luban Town, Renhuai City, Zunyi City, Guizhou Province, China** |
| 93 | **Hongyingzi Genetic Variant** | **Luban Town, Renhuai City, Zunyi City, Guizhou Province, China** |
| 94 | **Hongyingzi Genetic Variant** | **Luban Town, Renhuai City, Zunyi City, Guizhou Province, China** |
| 95 | **Hongyingzi Genetic Variant** | **Daba Town, Renhuai City, Zunyi City, Guizhou Province, China** |
