# Supplementary material for: Evaluation of cold tolerance in sorghum germplasm from the Chishui River Basin in China: insights from germination, field trials, and physiological assays
Source: Front Plant Sci. 2025 Sep 2;16:1630271. doi: 10.3389/fpls.2025.1630271 (PMC12436481; doi:10.3389/fpls.2025.1630271)
Supplement: Supplementary file 2 [file Table2.doc]

Supplementary Table 2. Comparison of germination indices of sorghum resources at different temperatures.

| Material | **Temperature** | GPo（%） | GPe（%） | RL(cm) | PL(cm) | RFW(mg) | PFW(mg) |
| --- | --- | --- | --- | --- | --- | --- | --- |
| 1 | 25℃ | 50.67±1.33 | 96.00±2.31 | 5.63±0.22 | 6.70±0.56 | 41.83±4.39 | 17.17±1.04 |
|  | 15℃ | 8±2.31 | 30.67±1.33 | 1.15±0.03 | 2.50±0.15 | 2.77±0.33 | 4.27±0.03 |
|  | 10℃ | 0.00±0.00 | 20.00±2.31 | 0.77±0.07 | 1.77±0.15 | 5.60±0.62 | 8.43±1.17 |
| 2 | 25℃ | 58.67±1.33 | 97.33±2.67 | 8.53±0.34 | 11.90±1.05 | 62.90±2.55 | 43.17±5.50 |
|  | 15℃ | 26.67±1.33 | 63.33±1.76 | 2.20±0.00 | 3.60±0.21 | 4.33±0.09 | 17.37±0.27 |
|  | 10℃ | 0.00±0.00 | 40.00±4.00 | 2.07±0.09 | 4.30±0.12 | 12.57±0.18 | 25.03±1.45 |
| 3 | 25℃ | 90.67±1.33 | 98.67±1.33 | 7.43±0.17 | 11.40±1.05 | 66.80±0.93 | 48.30±0.89 |
|  | 15℃ | 36.00±1.15 | 82.00±1.15 | 2.20±0.00 | 3.40±0.34 | 5.42±0.17 | 17.93±0.21 |
|  | 10℃ | 0.00±0.00 | 48.00±2.31 | 2.60±0.21 | 5.67±0.23 | 10.47±0.37 | 25.87±2.03 |
| 4 | 25℃ | 78.67±4.81 | 92.00±2.31 | 5.63±0.22 | 6.70±0.56 | 38.83±1.59 | 17.17±1.04 |
|  | 15℃ | 13.33±2.67 | 45.33±1.76 | 1.27±0.33 | 2.67±0.15 | 2.50±0.17 | 4.57±0.09 |
|  | 10℃ | 0.00±0.00 | 32.00±0.00 | 1.93±0.09 | 4.50±0.12 | 5.63±0.35 | 13.100.52 |
| 5 | 25℃ | 57.33±1.33 | 90.67±1.33 | 7.87±0.18 | 11.53±0.35 | 63.93±1.29 | 34.07±1.02 |
|  | 15℃ | 30.67±4.81 | 57.33±2.67 | 1.24±0.03 | 2.83±0.26 | 2.77±0.12 | 4.90±0.47 |
|  | 10℃ | 0.00±0.00 | 42.67±1.33 | 0.73±0.03 | 2.47±0.07 | 2.90±0.26 | 5.36±0.37 |
| 6 | 25℃ | 76.00±2.31 | 98.67±1.33 | 6.97±0.09 | 9.23±0.88 | 54.13±1.40 | 47.67±0.77 |
|  | 15℃ | 13.33±2.67 | 54.67±3.53 | 1.71±0.10 | 2.87±0.09 | 6.17±0.43 | 7.89±0.22 |

（Continued from Supplementary Table 2）

| 6 | 10℃ | 0.00±0.00 | 30.67±1.33 | 0.57±0.03 | 3.47±0.18 | 0.97±0.15 | 6.13±0.32 |
| --- | --- | --- | --- | --- | --- | --- | --- |
| 7 | 25℃ | 73.33±5.33 | 80.00±2.31 | 8.03±0.41 | 11.27±0.88 | 56.17±3.07 | 34.77±1.74 |
|  | 15℃ | 34.67±1.33 | 53.33±2.67 | 1.77±0.37 | 4.17±0.47 | 5.27±0.77 | 7.40±0.98 |
|  | 10℃ | 0.00±0.00 | 42.67±2.67 | 2.00±0.06 | 4.37±0.07 | 7.60±0.15 | 18.43±0.60 |
| 8 | 25℃ | 88.00±2.31 | 96.00±2.31 | 6.07±0.15 | 10.63±0.64 | 41.93±0.66 | 30.20±2.31 |
|  | 15℃ | 30.67±2.67 | 65.33±2.67 | 1.60±0.15 | 2.97±0.18 | 4.53±0.20 | 5.23±0.20 |
|  | 10℃ | 0.00±0.00 | 41.33±2.67 | 2.03±0.07 | 4.47±0.09 | 8.17±0.50 | 19.90±0.11 |
| 9 | 25℃ | 92.00±2.31 | 97.33±2.67 | 7.07±0.09 | 12.03±0.79 | 60.27±3.83 | 40.60±2.51 |
|  | 15℃ | 32.00±2.31 | 56.00±4.62 | 1.97±0.07 | 4.87±0.39 | 8.47±0.36 | 9.73±0.85 |
|  | 10℃ | 0.00±0.00 | 50.67±1.33 | 1.57±0.19 | 4.13±0.32 | 8.77±0.43 | 17.17±1.69 |
| 10 | 25℃ | 90.67±7.42 | 92.00±2.31 | 8.70±0.36 | 11.43±0.44 | 62.13±4.07 | 29.07±0.81 |
|  | 15℃ | 37.33±1.33 | 52.00±4.62 | 1.27±0.18 | 4.23±0.09 | 9.73±0.20 | 10.80±0.35 |
|  | 10℃ | 0.00±0.00 | 52.00±0.00 | 2.00±0.10 | 4.57±0.03 | 6.37±1.24 | 13.63±1.30 |
| 11 | 25℃ | 86.67±1.33 | 93.33±1.33 | 4.97±0.18 | 7.00±0.35 | 46.90±2.49 | 33.90±1.21 |
|  | 15℃ | 4.00±2.31 | 45.33±2.67 | 1.20±0.06 | 3.57±0.03 | 3.33±0.03 | 6.23±0.22 |
|  | 10℃ | 0.00±0.00 | 30.67±1.33 | 0.77±0.03 | 2.73±0.18 | 5.23±0.66 | 6.47±0.70 |
| 12 | 25℃ | 82.67±1.33 | 98.67±1.33 | 7.03±0.12 | 11.10±0.40 | 59.47±4.63 | 41.47±2.65 |
|  | 15℃ | 46.33±1.20 | 74.67±1.33 | 2.37±0.18 | 7.87±0.39 | 10.20±0.06 | 17.33±0.12 |
|  | 10℃ | 0.00±0.00 | 58.67±3.53 | 2.63±0.07 | 4.30±0.10 | 12.70±0.26 | 15.77±0.09 |

**（Continued from Supplementary Table 2）**

| 13 | 25℃ | 82.67±3.53 | 97.33±1.33 | 8.30±0.15 | 9.40±0.20 | 54.03±0.52 | 22.97±0.94 |
| --- | --- | --- | --- | --- | --- | --- | --- |
|  | 15℃ | 28.00±0.00 | 75.33±0.67 | 1.90±0.10 | 4.13±0.48 | 7.21±0.51 | 8.03±1.48 |
|  | 10℃ | 0.00±0.00 | 57.33±1.33 | 1.53±0.19 | 2.97±0.07 | 10.40±1.37 | 13.33±0.21 |
| 14 | 25℃ | 72.00±2.31 | 86.67±3.53 | 7.27±0.12 | 9.70±0.26 | 55.83±0.79 | 19.10±0.36 |
|  | 15℃ | 14.67±2.67 | 50.00±5.03 | 1.02±0.09 | 2.93±0.41 | 2.57±0.39 | 4.37±0.27 |
|  | 10℃ | 0.00±0.00 | 41.33±2.67 | 1.27±0.12 | 2.23±0.15 | 5.20±1.10 | 8.43±0.68 |
| 15 | 25℃ | 62.67±2.67 | 66.67±1.33 | 5.37±0.32 | 7.23±0.13 | 34.33±1.41 | 15.97±1.22 |
|  | 15℃ | 12.00±2.31 | 28.00±4.62 | 1.30±0.06 | 2.31±0.36 | 4.37±0.17 | 4.96±0.44 |
|  | 10℃ | 0.00±0.00 | 20.00±4.00 | 0.73±0.48 | 2.00±0.06 | 0.67±0.47 | 1.47±0.20 |
| 16 | 25℃ | 52.00±6.11 | 65.33±5.81 | 6.17±0.07 | 9.63±0.09 | 40.67±3.41 | 15.10±0.23 |
|  | 15℃ | 13.33±1.33 | 20.00±2.31 | 0.90±0.15 | 1.74±0.18 | 2.33±0.28 | 4.43±0.44 |
|  | 10℃ | 0.00±0.00 | 25.33±1.33 | 1.36±0.13 | 2.70±0.06 | 11.10±0.51 | 14.57±0.50 |
| 17 | 25℃ | 48.00±8.00 | 60.00±4.00 | 6.10±0.50 | 8.23±0.64 | 50.70±2.21 | 19.03±0.30 |
|  | 15℃ | 2.67±2.67 | 29.33±1.33 | 0.59±0.01 | 1.34±0.13 | 2.42±0.02 | 3.13±0.38 |
|  | 10℃ | 0.00±0.00 | 17.33±1.33 | 0.26±0.03 | 1.50±0.06 | 0.10±0.00 | 6.23±0.23 |
| 18 | 25℃ | 37.33±1.33 | 69.33±1.33 | 4.40±0.12 | 7.27±0.52 | 24.10±0.64 | 33.23±1.17 |
|  | 15℃ | 1.33±1.33 | 10.67±3.53 | 1.14±0.07 | 2.37±0.18 | 4.45±0.38 | 7.39±0.70 |
|  | 10℃ | 0.00±0.00 | 7.00±1.00 | 0.70±0.21 | 1.23±0.09 | 2.17±0.03 | 4.60±0.15 |
| 19 | 25℃ | 13.33±1.33 | 51.33±2.40 | 4.17±0.22 | 6.37±0.20 | 30.57±0.90 | 12.93±0.32 |

（Continued from Supplementary Table 2）

| 19 | 15℃ | 4.00±2.31 | 40.00±1.15 | 1.30±0.12 | 2.50±0.00 | 4.53±0.23 | 5.93±0.27 |
| --- | --- | --- | --- | --- | --- | --- | --- |
|  | 10℃ | 0.00±0.00 | 29.33±1.33 | 0.60±0.25 | 5.50±0.21 | 1.07±0.21 | 8.97±0.14 |
| 20 | 25℃ | 22.67±0.66 | 55.00±5.00 | 4.17±0.22 | 6.37±0.20 | 31.23±0.26 | 25.93±0.31 |
|  | 15℃ | 1.00±1.00 | 16.67±0.67 | 1.10±0.15 | 3.53±0.22 | 2.37±0.22 | 6.37±0.18 |
|  | 10℃ | 0.00±0.00 | 30.67±1.33 | 0.37±0.08 | 2.40±0.30 | 1.00±0.00 | 4.17±0.12 |
| 21 | 25℃ | 16.00±2.31 | 60.67±1.76 | 4.13±0.18 | 5.03±0.17 | 28.43±4.89 | 25.10±0.32 |
|  | 15℃ | 5.33±1.33 | 50.67±3.71 | 1.77±0.03 | 4.40±0.15 | 4.03±0.29 | 7.90±0.61 |
|  | 10℃ | 0.00±0.00 | 41.33±1.33 | 0.70±0.12 | 2.80±0.25 | 3.43±0.03 | 6.63±0.54 |
| 22 | 25℃ | 64.00±2.31 | 81.33±1.33 | 7.23±0.32 | 10.23±1.27 | 53.07±3.22 | 21.43±1.32 |
|  | 15℃ | 30.67±0.67 | 53.33±0.67 | 1.60±0.12 | 4.77±0.13 | 9.78±0.77 | 15.20±0.52 |
|  | 10℃ | 0.00±0.00 | 49.33±1.33 | 2.50±0.15 | 6.27±0.09 | 11.30±0.11 | 16.13±0.13 |
| 23 | 25℃ | 42.67±1.33 | 85.33±3.53 | 4.97±0.32 | 6.10±0.15 | 27.03±2.57 | 27.03±0.33 |
|  | 15℃ | 5.33±1.33 | 47.33±0.67 | 1.53±0.07 | 4.50±0.17 | 3.47±0.26 | 14.33±0.15 |
|  | 10℃ | 0.00±0.00 | 22.67±2.67 | 1.53±0.12 | 3.57±0.24 | 11.33±0.74 | 13.26±0.63 |
| 24 | 25℃ | 37.33±1.33 | 69.33±1.33 | 4.17±0.22 | 6.37±0.71 | 30.57±0.90 | 5.27±0.75 |
|  | 15℃ | 6.67±1.33 | 48.00±2.31 | 1.33±0.07 | 3.20±0.12 | 4.64±0.12 | 8.36±0.18 |
|  | 10℃ | 0.00±0.00 | 26.67±2.67 | 0.43±0.03 | 3.47±0.19 | 1.19±0.06 | 5.50±1.76 |
| 25 | 25℃ | 44.00±2.31 | 61.33±1.33 | 4.63±0.32 | 5.73±0.03 | 28.67±0.72 | 16.30±0.15 |
|  | 15℃ | 17.33±2.31 | 44.00±2.31 | 1.16±0.10 | 3.27±0.22 | 4.83±0.61 | 8.33±0.20 |

**（Continued from Supplementary Table 2）**

| 25 | 10℃ | 0.00±0.00 | 44.00±2.31 | 1.03±0.09 | 3.30±0.17 | 2.38±0.18 | 8.70±0.15 |
| --- | --- | --- | --- | --- | --- | --- | --- |
| 26 | 25℃ | 37.33±1.33 | 90.67±1.33 | 4.50±0.06 | 5.27±0.27 | 26.03±0.54 | 25.10±0.15 |
|  | 15℃ | 13.33±1.33 | 26.67±4.81 | 1.13±0.03 | 3.67±0.27 | 3.73±0.35 | 5.70±0.36 |
|  | 10℃ | 0.00±0.00 | 16.00±0.00 | 0.26±0.03 | 2.53±0.09 | 1.10±0.00 | 2.80±0.40 |
| 27 | 25℃ | 80.00±4.00 | 92.00±2.31 | 6.07±0.15 | 10.63±0.64 | 42.27±0.66 | 32.20±2.70 |
|  | 15℃ | 22.67±2.67 | 29.33±1.33 | 1.07±0.03 | 3.57±0.29 | 4.83±0.49 | 5.63±0.38 |
|  | 10℃ | 0.00±0.00 | 33.33±1.33 | 0.93±0.15 | 1.37±0.09 | 2.57±0.81 | 5.36±0.26 |
| 28 | 25℃ | 42.67±1.33 | 85.33±3.53 | 6.10±0.25 | 9.43±0.61 | 53.10±1.97 | 31.53±0.72 |
|  | 15℃ | 9.33±3.59 | 25.33±1.33 | 0.90±0.06 | 3.53±0.12 | 3.73±0.03 | 6.83±0.12 |
|  | 10℃ | 0.00±0.00 | 24.00±4.00 | 0.63±0.12 | 3.37±0.46 | 1.97±0.41 | 4.93±0.41 |
| 30 | 25℃ | 13.33±1.33 | 54.67±2.67 | 3.67±0.19 | 5.47±0.23 | 31.67±0.32 | 15.43±0.03 |
|  | 15℃ | 4.67±0.67 | 24.00±8.00 | 1.10±0.10 | 2.93±0.09 | 3.43±0.20 | 5.93±0.32 |
|  | 10℃ | 0.00±0.00 | 22.67±3.53 | 0.47±0.03 | 2.90±0.06 | 5.77±0.67 | 7.53±0.33 |
| 32 | 25℃ | 28.67±0.66 | 54.67±2.67 | 5.13±0.22 | 7.60±0.10 | 41.10±1.04 | 14.97±0.18 |
|  | 15℃ | 7.67±0.33 | 33.33±2.91 | 1.53±0.18 | 3.50±0.00 | 3.95±0.16 | 5.40±0.15 |
|  | 10℃ | 0.00±0.00 | 14.67±3.53 | 1.14±0.09 | 2.77±0.27 | 3.06±0.77 | 4.67±0.56 |
| 38 | 25℃ | 88.00±2.31 | 93.33±2.67 | 7.33±0.07 | 10.77±0.26 | 63.80±1.65 | 31.70±1.20 |
|  | 15℃ | 49.33±1.33 | 54.67±2.67 | 2.40±0.21 | 5.53±0.18 | 5.23±0.24 | 10.03±0.38 |
|  | 10℃ | 0.00±0.00 | 49.33±3.53 | 1.37±0.18 | 5.13±0.37 | 7.07±1.16 | 10.76±1.75 |

**（Continued from Supplementary Table 2）**

| 39 | 25℃ | 82.67±1.33 | 93.33±2.67 | 6.20±0.23 | 10.20±0.15 | 53.83±2.03 | 30.80±4.09 |
| --- | --- | --- | --- | --- | --- | --- | --- |
|  | 15℃ | 20.00±4.00 | 52.00±4.62 | 2.73±0.03 | 6.00±0.45 | 7.93±0.77 | 13.17±0.37 |
|  | 10℃ | 0.00±0.00 | 40.00±2.31 | 0.50±0.06 | 1.73±0.29 | 2.37±0.12 | 4.10±0.31 |
| 40 | 25℃ | 76.00±2.31 | 81.33±1.33 | 3.50±0.10 | 4.60±0.06 | 31.10±0.17 | 12.73±0.53 |
|  | 15℃ | 9.33±1.33 | 33.33±1.33 | 1.57±0.03 | 2.37±0.12 | 3.54±0.18 | 6.20±0.10 |
|  | 10℃ | 0.00±0.00 | 18.67±3.53 | 1.00±0.15 | 3.13±0.32 | 2.47±0.67 | 5.60±1.01 |
| 41 | 25℃ | 84.00±2.31 | 93.33±1.33 | 5.43±0.09 | 7.93±0.39 | 44.53±1.88 | 19.20±1.96 |
|  | 15℃ | 0.00±0.00 | 33.33±1.33 | 1.53±0.18 | 3.90±0.12 | 12.07±0.56 | 8.43±0.23 |
|  | 10℃ | 0.00±0.00 | 24.00±2.31 | 0.97±0.19 | 3.06±0.09 | 3.20±0.40 | 7.00±0.32 |
| 42 | 25℃ | 65.33±1.33 | 77.33±1.33 | 4.47±0.23 | 8.07±0.93 | 45.83±4.08 | 23.33±2.46 |
|  | 15℃ | 8.00±0.00 | 45.33±1.33 | 1.57±0.03 | 4.13±0.29 | 3.80±0.15 | 7.10±0.25 |
|  | 10℃ | 0.00±0.00 | 34.67±4.81 | 0.97±0.03 | 3.50±0.20 | 2.30±0.53 | 5.43±0.84 |
| 43 | 25℃ | 30.67±2.67 | 54.67±2.67 | 3.40±0.10 | 4.90±0.35 | 27.93±1.40 | 12.23±0.52 |
|  | 15℃ | 12.00±0.00 | 28.00±2.31 | 1.30±0.06 | 2.40±0.21 | 3.61±0.20 | 7.20±0.12 |
|  | 10℃ | 0.00±0.00 | 21.33±2.67 | 0.83±0.12 | 3.07±0.13 | 2.50±0.17 | 6.13±0.48 |
| 44 | 25℃ | 33.33±4.81 | 78.67±5.81 | 4.67±0.12 | 7.00±0.46 | 32.53±4.65 | 13.20±2.15 |
|  | 15℃ | 5.33±1.33 | 25.33±2.67 | 0.43±0.09 | 1.10±0.10 | 1.50±0.26 | 3.87±0.60 |
|  | 10℃ | 0.00±0.00 | 17.33±3.53 | 0.10±0.00 | 1.13±0.19 | 0.17±0.03 | 0.56±0.09 |

（Continued from Supplementary Table 2）

| 45 | 25℃ | 19.00±1.53 | 42.67±2.67 | 4.10±0.15 | 6.30±0.21 | 27.83±2.56 | 15.83±0.34 |
| --- | --- | --- | --- | --- | --- | --- | --- |
|  | 15℃ | 13.33±1.33 | 34.67±1.33 | 1.39±0.26 | 3.23±0.22 | 3.00±0.51 | 7.30±1.31 |
|  | 10℃ | 0.00±0.00 | 20.00±2.31 | 1.33±0.15 | 4.10±0.29 | 3.23±0.42 | 7.23±0.31 |
| 46 | 25℃ | 32.00±2.31 | 56.67±2.41 | 5.50±0.15 | 9.37±0.27 | 43.80±3.79 | 26.43±0.64 |
|  | 15℃ | 10.67±1.33 | 45.33±2.67 | 0.73±0.09 | 1.53±0.07 | 2.63±0.03 | 4.23±0.12 |
|  | 10℃ | 0.00±0.00 | 29.33±2.67 | 0.53±0.12 | 2.00±0.21 | 3.13±0.33 | 7.10±0.26 |
| 47 | 25℃ | 48.00±8.00 | 60.00±4.62 | 4.13±0.18 | 5.37±0.44 | 28.43±4.89 | 24.77±0.43 |
|  | 15℃ | 4.00±2.31 | 20.00±2.31 | 0.45±0.03 | 1.57±0.19 | 1.45±0.03 | 2.46±0.19 |
|  | 10℃ | 0.00±0.00 | 6.67±1.33 | 0.80±0.10 | 1.80±0.06 | 4.86±0.88 | 5.80±0.00 |
| 48 | 25℃ | 33.33±4.81 | 88.00±2.31 | 5.70±0.82 | 8.07±0.90 | 48.53±2.75 | 24.00±1.93 |
|  | 15℃ | 14.67±1.33 | 41.33±3.53 | 0.77±0.09 | 2.23±0.09 | 1.16±0.38 | 2.25±0.23 |
|  | 10℃ | 0.00±0.00 | 29.33±1.33 | 0.90±0.12 | 2.17±0.03 | 4.47±0.63 | 6.40±0.79 |
| 49 | 25℃ | 58.67±5.81 | 66.67±1.33 | 5.70±0.82 | 8.73±1.49 | 48.46±3.94 | 25.33±3.04 |
|  | 15℃ | 27.33±0.67 | 46.00±1.15 | 0.85±0.05 | 2.17±0.15 | 2.50±0.14 | 3.57±0.07 |
|  | 10℃ | 0.00±0.00 | 50.67±2.67 | 1.31±0.12 | 3.63±0.14 | 3.60±0.64 | 7.40±0.50 |
| 50 | 25℃ | 70.67±3.53 | 80.00±2.31 | 6.17±0.03 | 10.73±0.54 | 64.87±2.60 | 39.43±1.09 |
|  | 15℃ | 0.00±0.00 | 57.33±4.81 | 0.77±0.09 | 1.83±0.19 | 2.27±0.09 | 2.71±0.07 |
|  | 10℃ | 0.00±0.00 | 30.67±2.67 | 0.65±0.03 | 1.57±0.09 | 2.13±0.03 | 5.46±0.44 |
| 51 | 25℃ | 42.67±2.67 | 66.67±4.81 | 5.80±0.06 | 6.90±0.06 | 55.63±1.09 | 34.83±0.44 |

**（Continued from Supplementary Table 2）**

| 51 | 15℃ | 5.33±1.33 | 34.67±1.33 | 0.63±0.13 | 1.63±0.23 | 1.74±0.09 | 2.77±0.30 |
| --- | --- | --- | --- | --- | --- | --- | --- |
|  | 10℃ | 0.00±0.00 | 30.67±1.33 | 0.53±0.12 | 1.87±0.07 | 2.45±0.33 | 6.73±0.06 |
| 52 | 25℃ | 56.00±2.31 | 88.00±2.31 | 6.10±0.23 | 7.33±0.38 | 59.17±2.85 | 43.80±1.56 |
|  | 15℃ | 6.67±3.53 | 34.67±0.67 | 0.67±0.07 | 2.37±0.12 | 2.17±0.03 | 3.50±0.50 |
|  | 10℃ | 0.00±0.00 | 44.00±2.31 | 0.30±0.06 | 1.83±0.07 | 1.27±0.73 | 3.66±0.79 |
| 53 | 25℃ | 50.67±1.33 | 84.00±2.31 | 3.97±0.07 | 6.40±0.25 | 26.50±0.65 | 12.53±0.52 |
|  | 15℃ | 13.33±1.33 | 42.67±1.33 | 1.43±0.18 | 2.87±0.39 | 3.67±0.26 | 6.30±0.17 |
|  | 10℃ | 0.00±0.00 | 24.00±0.00 | 0.33±0.03 | 2.00±0.42 | 2.50±0.30 | 4.73±0.34 |
| 67 | 25℃ | 82.67±4.81 | 89.33±3.53 | 8.30±0.31 | 12.17±0.72 | 67.30±2.49 | 42.57±3.05 |
|  | 15℃ | 10.67±1.33 | 24.00±2.31 | 1.23±0.13 | 2.13±0.03 | 3.36±0.17 | 4.56±0.23 |
|  | 10℃ | 0.00±0.00 | 21.33±1.33 | 0.47±0.09 | 1.73±0.09 | 2.43±0.38 | 4.20±0.87 |
| 68 | 25℃ | 82.67±4.81 | 89.33±3.53 | 6.87±0.15 | 9.27±0.69 | 59.93±7.43 | 30.53±5.47 |
|  | 15℃ | 6.67±3.53 | 45.33±2.67 | 1.77±0.33 | 3.40±0.42 | 3.03±0.39 | 6.33±0.65 |
|  | 10℃ | 0.00±0.00 | 30.67±1.33 | 0.60±0.17 | 2.63±0.27 | 3.33±0.67 | 6.40±0.34 |
| 73 | 25℃ | 54.67±1.33 | 70.67±1.33 | 5.40±0.21 | 9.13±0.18 | 40.27±1.08 | 21.07±0.49 |
|  | 15℃ | 2.67±2.67 | 48.00±0.00 | 0.86±0.02 | 2.00±0.10 | 3.27±0.20 | 4.73±0.39 |
|  | 10℃ | 0.00±0.00 | 36.00±0.00 | 0.33±0.03 | 1.77±0.09 | 2.03±0.29 | 3.96±0.23 |
| 74 | 25℃ | 56.00±2.31 | 69.33±1.33 | 5.50±0.20 | 8.23±0.20 | 40.70±3.84 | 33.50±3.80 |
|  | 15℃ | 21.33±7.42 | 56.00±2.31 | 1.27±0.09 | 3.63±0.15 | 2.57±0.20 | 4.56±0.12 |

**（Continued from Supplementary Table 2）**

| 74 | 10℃ | 0.00±0.00 | 33.33±1.33 | 0.27±0.06 | 1.80±0.06 | 1.48±0.13 | 3.90±0.15 |
| --- | --- | --- | --- | --- | --- | --- | --- |
| 75 | 25℃ | 49.33±4.81 | 62.67±2.67 | 6.00±0.26 | 8.93±0.28 | 51.30±1.45 | 14.03±0.52 |
|  | 15℃ | 26.67±1.33 | 53.33±3.53 | 1.57±0.20 | 3.63±0.34 | 3.53±0.20 | 7.63±0.58 |
|  | 10℃ | 0.00±0.00 | 37.33±1.33 | 1.36±0.13 | 2.70±0.06 | 11.10±0.51 | 9.57±0.14 |
| 76 | 25℃ | 65.33±1.33 | 76.00±2.31 | 5.13±0.22 | 7.60±0.10 | 42.77±2.59 | 34.50±0.74 |
|  | 15℃ | 14.67±1.33 | 33.33±1.33 | 1.60±0.21 | 4.87±0.12 | 4.03±0.24 | 8.27±0.38 |
|  | 10℃ | 0.00±0.00 | 14.67±3.53 | 0.86±0.03 | 2.17±0.12 | 4.23±0.44 | 5.90±0.25 |
| 77 | 25℃ | 69.33±2.67 | 77.33±4.81 | 7.27±0.38 | 9.87±0.52 | 59.07±3.42 | 34.83±2.37 |
|  | 15℃ | 21.33±5.33 | 36.00±2.31 | 0.97±0.23 | 2.23±0.50 | 3.17±0.28 | 3.10±0.44 |
|  | 10℃ | 0.00±0.00 | 18.67±1.33 | 0.33±0.03 | 1.77±0.09 | 2.03±0.29 | 3.96±0.23 |
| 78 | 25℃ | 50.67±1.33 | 77.33±3.53 | 6.40±0.26 | 9.63±0.67 | 47.70±1.36 | 23.17±1.38 |
|  | 15℃ | 8.00±0.00 | 25.33±2.67 | 0.77±0.09 | 2.10±0.17 | 2.19±0.15 | 2.96±0.47 |
|  | 10℃ | 0.00±0.00 | 14.67±1.33 | 0.73±0.13 | 2.20±0.15 | 3.70±0.45 | 6.43±0.38 |
| 79 | 25℃ | 80.00±0.00 | 93.33±1.33 | 8.10±0.35 | 11.43±0.56 | 58.77±1.56 | 12.87±0.46 |
|  | 15℃ | 12.00±2.31 | 18.67±1.33 | 0.87±0.09 | 1.53±0.07 | 1.73±0.09 | 3.57±0.12 |
|  | 10℃ | 0.00±0.00 | 10.67±1.33 | 0.77±0.07 | 1.67±0.27 | 3.03±0.34 | 6.03±0.59 |
| 80 | 25℃ | 53.33±3.53 | 66.67±1.33 | 7.70±0.06 | 11.10±0.58 | 65.10±0.36 | 15.20±0.30 |
|  | 15℃ | 14.67±1.33 | 49.33±1.33 | 0.80±0.06 | 2.33±0.18 | 4.13±0.03 | 6.51±0.34 |
|  | 10℃ | 0.00±0.00 | 32.00±8.00 | 0.23±0.03 | 1.57±0.09 | 0.80±0.05 | 2.83±0.26 |

（Continued from Supplementary Table 2）

| 81 | 25℃ | 53.33±1.33 | 85.33±1.33 | 6.50±0.15 | 8.97±0.91 | 40.13±0.23 | 12.90±0.98 |
| --- | --- | --- | --- | --- | --- | --- | --- |
|  | 15℃ | 29.33±1.33 | 40.00±0.00 | 1.47±0.03 | 4.73±0.12 | 3.43±0.19 | 6.13±0.23 |
|  | 10℃ | 0.00±0.00 | 25.33±2.67 | 0.43±0.09 | 2.20±0.35 | 2.03±0.33 | 4.76±0.38 |
| 82 | 25℃ | 70.67±3.53 | 80.00±2.31 | 7.33±0.07 | 11.43±0.90 | 65.13±0.32 | 32.70±0.20 |
|  | 15℃ | 32.00±2.31 | 48.00±6.11 | 1.20±0.06 | 3.43±0.61 | 3.10±0.12 | 5.30±0.47 |
|  | 10℃ | 0.00±0.00 | 33.33±2.67 | 0.30±0.06 | 1.73±0.22 | 2.27±0.82 | 4.50±0.25 |
| 83 | 25℃ | 60.00±2.31 | 73.33±2.67 | 6.70±0.10 | 10.00±0.80 | 46.93±2.71 | 21.23±0.48 |
|  | 15℃ | 42.67±3.53 | 53.33±1.33 | 1.33±0.13 | 3.37±0.09 | 2.77±0.43 | 4.30±0.31 |
|  | 10℃ | 0.00±0.00 | 50.67±1.33 | 0.57±0.03 | 3.03±0.09 | 4.00±0.97 | 6.36±0.41 |
| 84 | 25℃ | 54.67±1.33 | 84.00±2.31 | 6.30±0.12 | 7.93±0.34 | 41.03±0.09 | 11.30±0.15 |
|  | 15℃ | 20.00±0.00 | 26.67±1.33 | 0.87±0.03 | 2.27±0.29 | 2.25±0.15 | 3.16±0.58 |
|  | 10℃ | 0.00±0.00 | 18.67±1.33 | 0.67±0.13 | 1.87±0.28 | 3.37±0.39 | 7.03±0.29 |
| 85 | 25℃ | 78.67±1.33 | 89.33±1.33 | 9.10±0.90 | 13.33±1.45 | 53.17±5.35 | 13.47±1.84 |
|  | 15℃ | 26.67±3.53 | 46.67±2.67 | 1.17±0.03 | 3.57±0.03 | 4.83±0.07 | 7.16±0.11 |
|  | 10℃ | 0.00±0.00 | 25.33±2.67 | 0.33±0.09 | 1.67±0.03 | 0.97±1.17 | 3.93±0.18 |
| 86 | 25℃ | 58.67±1.33 | 77.33±1.33 | 7.37±0.23 | 10.80±1.47 | 58.80±3.00 | 35.00±1.22 |
|  | 15℃ | 7.33±0.67 | 54.67±1.33 | 0.96±0.07 | 1.97±0.23 | 1.43±0.12 | 2.53±0.09 |
|  | 10℃ | 0.00±0.00 | 37.33±4.81 | 1.20±0.12 | 3.27±0.17 | 8.47±0.54 | 12.30±0.47 |

（Continued from Supplementary Table 2）

| 87 | 25℃ | 50.67±2.67 | 65.33±2.67 | 6.60±0.10 | 9.53±0.66 | 48.53±0.56 | 14.47±0.83 |
| --- | --- | --- | --- | --- | --- | --- | --- |
|  | 15℃ | 24.00±0.00 | 48.00±2.31 | 0.77±0.06 | 2.70±0.23 | 1.50±0.06 | 3.23±0.57 |
|  | 10℃ | 0.00±0.00 | 30.67±1.33 | 1.03±0.19 | 2.63±0.33 | 5.27±1.42 | 5.57±0.21 |
| 88 | 25℃ | 69.33±1.33 | 78.67±1.33 | 9.83±0.38 | 13.00±0.66 | 57.67±0.50 | 34.33±0.71 |
|  | 15℃ | 22.67±1.33 | 66.00±2.00 | 0.67±0.09 | 1.77±0.29 | 1.80±0.06 | 2.80±0.38 |
|  | 10℃ | 0.00±0.00 | 40.00±2.31 | 0.70±0.12 | 2.97±0.18 | 4.43±0.22 | 6.43±0.79 |
| 89 | 25℃ | 65.33±1.33 | 80.00±2.31 | 8.97±0.29 | 12.63±0.54 | 63.80±1.56 | 41.90±0.78 |
|  | 15℃ | 21.33±1.33 | 45.33±2.67 | 1.48±0.21 | 4.57±0.03 | 5.87±0.34 | 8.16±0.23 |
|  | 10℃ | 0.00±0.00 | 38.67±1.33 | 0.58±0.11 | 2.50±0.15 | 2.97±1.22 | 4.93±1.03 |
| 90 | 25℃ | 70.67±1.33 | 80.00±2.31 | 8.43±0.17 | 8.93±0.13 | 79.73±0.27 | 40.03±1.23 |
|  | 15℃ | 0.00±0.00 | 45.33±2.67 | 1.37±0.17 | 3.40±0.10 | 4.90±0.17 | 7.37±0.07 |
|  | 10℃ | 0.00±0.00 | 18.67±1.33 | 0.30±0.00 | 3.33±0.15 | 1.70±0.15 | 7.83±0.29 |
| 91 | 25℃ | 69.33±1.33 | 81.33±1.33 | 8.30±0.31 | 12.17±0.72 | 67.30±2.49 | 42.57±3.05 |
|  | 15℃ | 29.33±6.67 | 54.67±3.53 | 1.16±0.12 | 2.93±0.64 | 4.43±0.48 | 6.57±0.21 |
|  | 10℃ | 0.00±0.00 | 42.67±3.53 | 0.33±0.03 | 1.77±0.09 | 2.17±0.18 | 3.96±0.23 |
| 92 | 25℃ | 84.00±2.31 | 94.67±1.33 | 8.17±0.09 | 9.97±0.84 | 60.70±0.81 | 14.03±1.16 |
|  | 15℃ | 29.33±1.33 | 52.00±2.31 | 1.30±0.21 | 3.17±0.33 | 3.10±0.25 | 6.63±0.58 |
|  | 10℃ | 0.00±0.00 | 37.33±2.67 | 0.50±0.06 | 2.07±0.17 | 2.47±0.20 | 4.53±0.32 |
| 93 | 25℃ | 62.67±2.67 | 74.67±1.33 | 6.50±0.15 | 8.97±0.91 | 43.47±0.67 | 12.90±0.98 |

（Continued from Supplementary Table 2）

| 93 | 15℃ | 33.33±1.33 | 53.33±2.67 | 0.90±0.03 | 2.47±0.15 | 4.13±0.15 | 6.63±0.58 |
| --- | --- | --- | --- | --- | --- | --- | --- |
|  | 10℃ | 0.00±0.00 | 21.33±2.67 | 0.37±0.07 | 2.20±0.15 | 2.17±0.66 | 4.50±0.12 |
| 94 | 25℃ | 65.33±2.67 | 82.67±1.33 | 6.03±0.24 | 8.50±0.46 | 56.90±4.39 | 19.50±1.10 |
|  | 15℃ | 2.67±1.33 | 49.33±5.81 | 0.87±0.01 | 2.33±0.12 | 4.53±0.09 | 5.90±0.53 |
|  | 10℃ | 0.00±0.00 | 25.33±1.33 | 0.57±0.09 | 1.83±0.17 | 3.07±0.37 | 3.96±0.70 |
| 95 | 25℃ | 90.67±1.33 | 100.00±0.00 | 10.53±0.03 | 15.17±0.43 | 58.40±0.00 | 42.00±0.73 |
|  | 15℃ | 42.67±1.33 | 77.33±4.81 | 2.57±0.03 | 3.57±0.03 | 11.30±0.55 | 16.47±0.03 |
|  | 10℃ | 0.00±0.00 | 58.67±1.33 | 0.50±0.10 | 3.00±0.12 | 2.10±0.23 | 8.36±0.35 |

GPo - Germination potential, GPe - Germination percentage, PL - Plumule length, RL - Radicle length, PFW - Plumule fresh weight, RFW - Radicle fresh weight.
