# Supplementary material for: Evaluation of cold tolerance in sorghum germplasm from the Chishui River Basin in China: insights from germination, field trials, and physiological assays
Source: Front Plant Sci. 2025 Sep 2;16:1630271. doi: 10.3389/fpls.2025.1630271 (PMC12436481; doi:10.3389/fpls.2025.1630271)
Supplement: Supplementary file 3 [file Table3.doc]

Supplementary Table 3. Effects of temperature on germinaiton indicators.

| Indicator | Temperature(℃) | Max | Min | Average | SD | CV（%） |
| --- | --- | --- | --- | --- | --- | --- |
| GPo (%) | 25℃ | 100.00 | 12.00 | 59.73 | 21.07 | 35.28 |
|  | 15℃ | 52.00 | 0.00 | 17.10 | 12.91 | 75.50 |
|  | 10℃ | 0.00 | 0.00 | 0.00 | 0.00 | 0.00 |
| GPe (%) | 25℃ | 100.00 | 40.00 | 79.17 | 14.52 | 18.34 |
|  | 15℃ | 84.00 | 4.00 | 43.81 | 15.50 | 35.38 |
|  | 10℃ | 64.00 | 4.00 | 31.50 | 12.97 | 41.17 |
| SL (cm) | 25℃ | 10.90 | 3.30 | 6.28 | 1.64 | 26.11 |
|  | 15℃ | 2.80 | 0.30 | 1.29 | 0.52 | 40.31 |
|  | 10℃ | 2.90 | 0.10 | 0.88 | 0.63 | 71.59 |
| RL (cm) | 25℃ | 15.70 | 4.50 | 8.99 | 2.47 | 27.47 |
|  | 15℃ | 8.40 | 0.90 | 3.14 | 1.23 | 39.17 |
|  | 10℃ | 6.40 | 0.90 | 2.78 | 1.16 | 41.73 |
| PFW (mg) | 25℃ | 80.00 | 23.00 | 48.11 | 13.68 | 28.43 |
|  | 15℃ | 13.20 | 0.40 | 4.10 | 2.35 | 57.32 |
|  | 10℃ | 13.20 | 0.10 | 4.12 | 3.31 | 80.34 |
| RFW (mg) | 25℃ | 53.80 | 10.60 | 26.16 | 10.76 | 41.13 |
|  | 15℃ | 18.20 | 1.95 | 6.73 | 3.69 | 54.83 |
|  | 10℃ | 28.00 | 0.40 | 7.55 | 5.05 | 66.89 |
| R/P (length) | 25℃ | 1.96 | 1.02 | 1.44 | 0.21 | 14.58 |
|  | 15℃ | 4.63 | 1.14 | 2.54 | 0.66 | 25.98 |
|  | 10℃ | 19.33 | 1.00 | 4.31 | 2.72 | 63.11 |
| R/P (FW) | 25℃ | 1.50 | 0.20 | 0.56 | 0.22 | 39.29 |
|  | 15℃ | 4.88 | 0.61 | 1.75 | 0.65 | 37.14 |
|  | 10℃ | 19.00 | 0.49 | 2.64 | 1.36 | 51.52 |

GPo - Germination potential, GPe - Germination percentage, PL - Plumule length, RL - Radicle length, PFW - Plumule fresh weight, RFW - Radicle fresh weight, R/P(length)- Radicle to plumule ratio (length), R/P(FW)- Radicle to plumule ratio (fresh weight), Max - Maximum value, Min - Minimum value, SD - Standard deviation, CV - Coefficient of variation.
