# Supplementary material for: Evaluation of cold tolerance in sorghum germplasm from the Chishui River Basin in China: insights from germination, field trials, and physiological assays
Source: Front Plant Sci. 2025 Sep 2;16:1630271. doi: 10.3389/fpls.2025.1630271 (PMC12436481; doi:10.3389/fpls.2025.1630271)
Supplement: Supplementary file 4 [file Table4.doc]

Supplementary Table 4. Membership function analysis of sorghum germination characteristics under 10℃ cold stress treatment.

| Material | GPo | GPe | PL | RL | PFW | RFW | *D* | Sorting |
| --- | --- | --- | --- | --- | --- | --- | --- | --- |
| 1 | 0 | 0.165 | 0.328 | 0.193 | 0.314 | 0.619 | 0.270 | 30 |
| 2 | 0 | 0.470 | 0.626 | 0.324 | 0.472 | 0.757 | 0.441 | 13 |
| 3 | 0 | 0.585 | 0.931 | 0.499 | 0.368 | 0.677 | 0.510 | 6 |
| 4 | 0 | 0.376 | 0.913 | 0.748 | 0.342 | 1.000 | 0.563 | 2 |
| 5 | 0 | 0.562 | 0.203 | 0.117 | 0.104 | 0.154 | 0.190 | 46 |
| 6 | 0 | 0.319 | 0.170 | 0.342 | 0.038 | 0.113 | 0.163 | 53 |
| 7 | 0 | 0.658 | 0.650 | 0.356 | 0.319 | 0.670 | 0.442 | 11 |
| 8 | 0 | 0.503 | 0.889 | 0.400 | 0.458 | 0.857 | 0.518 | 4 |
| 9 | 0 | 0.639 | 0.568 | 0.299 | 0.346 | 0.532 | 0.397 | 20 |
| 10 | 0 | 0.706 | 0.593 | 0.368 | 0.235 | 0.582 | 0.414 | 16 |
| 11 | 0 | 0.346 | 0.378 | 0.360 | 0.265 | 0.200 | 0.258 | 33 |
| 12 | 0 | 0.750 | 1.000 | 0.352 | 0.509 | 0.465 | 0.513 | 5 |
| 13 | 0 | 0.741 | 0.460 | 0.254 | 0.453 | 0.741 | 0.442 | 12 |
| 14 | 0 | 0.569 | 0.431 | 0.139 | 0.217 | 0.546 | 0.317 | 25 |
| 15 | 0 | 0.305 | 0.327 | 0.200 | 0.041 | 0.061 | 0.156 | 59 |
| 16 | 0 | 0.449 | 0.568 | 0.206 | 0.657 | 0.506 | 0.398 | 19 |
| 17 | 0 | 0.291 | 0.066 | 0.076 | 0.000 | 0.059 | 0.082 | 69 |
| 18 | 0 | 0.000 | 0.383 | 0.060 | 0.209 | 0.127 | 0.130 | 65 |
| 19 | 0 | 0.720 | 0.332 | 1.000 | 0.078 | 0.895 | 0.504 | 7 |
| 20 | 0 | 0.703 | 0.195 | 0.334 | 0.071 | 0.157 | 0.244 | 36 |
| 21 | 0 | 0.881 | 0.416 | 0.578 | 0.298 | 0.300 | 0.412 | 17 |
| 22 | 0 | 0.767 | 0.916 | 0.679 | 0.505 | 0.982 | 0.642 | 1 |
| 23 | 0 | 0.254 | 0.813 | 0.620 | 1.000 | 0.613 | 0.550 | 3 |
| 24 | 0 | 0.432 | 0.237 | 0.585 | 0.089 | 0.236 | 0.263 | 31 |
| 25 | 0 | 0.935 | 0.571 | 0.605 | 0.193 | 0.673 | 0.496 | 8 |
| 26 | 0 | 0.115 | 0.106 | 0.482 | 0.096 | 0.090 | 0.148 | 61 |
| 27 | 0 | 0.398 | 0.378 | 0.001 | 0.138 | 0.167 | 0.180 | 49 |
| 28 | 0 | 0.271 | 0.232 | 0.316 | 0.084 | 0.151 | 0.176 | 50 |
| 30 | 0 | 0.488 | 0.303 | 0.547 | 0.428 | 0.610 | 0.396 | 21 |
| 32 | 0 | 0.254 | 0.567 | 0.320 | 0.175 | 0.367 | 0.281 | 28 |
| 38 | 0 | 0.648 | 0.468 | 0.470 | 0.257 | 0.400 | 0.374 | 22 |
| 39 | 0 | 0.498 | 0.170 | 0.057 | 0.100 | 0.126 | 0.159 | 57 |
| 40 | 0 | 0.195 | 0.754 | 0.748 | 0.184 | 0.538 | 0.403 | 18 |
| 41 | 0 | 0.237 | 0.442 | 0.353 | 0.165 | 0.456 | 0.276 | 29 |
| 42 | 0 | 0.530 | 0.557 | 0.438 | 0.112 | 0.269 | 0.318 | 24 |
| 43 | 0 | 0.449 | 0.640 | 0.678 | 0.210 | 0.609 | 0.431 | 14 |
| 44 | 0 | 0.193 | 0.000 | 0.052 | 0.009 | 0.000 | 0.042 | 71 |
| 45 | 0 | 0.555 | 0.869 | 0.713 | 0.270 | 0.567 | 0.496 | 9 |
| 46 | 0 | 0.629 | 0.217 | 0.115 | 0.170 | 0.307 | 0.240 | 37 |
| 47 | 0 | 0.022 | 0.491 | 0.285 | 0.455 | 0.259 | 0.252 | 34 |
| 48 | 0 | 0.353 | 0.426 | 0.200 | 0.212 | 0.317 | 0.251 | 35 |
| 49 | 0 | 1.000 | 0.607 | 0.419 | 0.176 | 0.345 | 0.425 | 15 |
| 50 | 0 | 0.432 | 0.239 | 0.024 | 0.074 | 0.126 | 0.149 | 60 |
| 51 | 0 | 0.556 | 0.199 | 0.192 | 0.100 | 0.202 | 0.208 | 40 |
| 52 | 0 | 0.606 | 0.078 | 0.166 | 0.044 | 0.053 | 0.158 | 58 |
| 53 | 0 | 0.281 | 0.178 | 0.255 | 0.219 | 0.457 | 0.232 | 38 |
| 67 | 0 | 0.209 | 0.097 | 0.019 | 0.081 | 0.074 | 0.080 | 70 |
| 68 | 0 | 0.371 | 0.185 | 0.212 | 0.126 | 0.254 | 0.191 | 45 |
| 73 | 0 | 0.621 | 0.115 | 0.089 | 0.116 | 0.196 | 0.189 | 47 |
| 74 | 0 | 0.576 | 0.084 | 0.123 | 0.085 | 0.103 | 0.162 | 55 |
| 75 | 0 | 0.756 | 0.586 | 0.236 | 0.512 | 0.880 | 0.495 | 10 |
| 76 | 0 | 0.143 | 0.420 | 0.213 | 0.233 | 0.172 | 0.197 | 42 |
| 77 | 0 | 0.217 | 0.069 | 0.069 | 0.078 | 0.093 | 0.088 | 68 |
| 78 | 0 | 0.134 | 0.261 | 0.140 | 0.180 | 0.319 | 0.172 | 51 |
| 79 | 0 | 0.020 | 0.210 | 0.027 | 0.118 | 0.581 | 0.159 | 56 |
| 80 | 0 | 0.569 | 0.025 | 0.017 | 0.024 | 0.194 | 0.138 | 63 |
| 81 | 0 | 0.299 | 0.128 | 0.164 | 0.116 | 0.448 | 0.192 | 43 |
| 82 | 0 | 0.478 | 0.055 | 0.038 | 0.078 | 0.125 | 0.129 | 66 |
| 83 | 0 | 0.899 | 0.179 | 0.241 | 0.194 | 0.350 | 0.311 | 27 |
| 84 | 0 | 0.184 | 0.241 | 0.144 | 0.190 | 0.795 | 0.259 | 32 |
| 85 | 0 | 0.279 | 0.046 | 0.000 | 0.040 | 0.359 | 0.121 | 67 |
| 86 | 0 | 0.579 | 0.402 | 0.245 | 0.342 | 0.421 | 0.332 | 23 |
| 87 | 0 | 0.563 | 0.383 | 0.202 | 0.255 | 0.469 | 0.312 | 26 |
| 88 | 0 | 0.620 | 0.141 | 0.135 | 0.178 | 0.194 | 0.211 | 39 |
| 89 | 0 | 0.582 | 0.124 | 0.094 | 0.106 | 0.097 | 0.167 | 52 |
| 90 | 0 | 0.203 | 0.040 | 0.331 | 0.046 | 0.206 | 0.138 | 64 |
| 91 | 0 | 0.646 | 0.052 | 0.025 | 0.072 | 0.066 | 0.143 | 62 |
| 92 | 0 | 0.446 | 0.113 | 0.109 | 0.092 | 0.394 | 0.192 | 44 |
| 93 | 0 | 0.282 | 0.099 | 0.169 | 0.115 | 0.423 | 0.181 | 48 |
| 94 | 0 | 0.313 | 0.204 | 0.122 | 0.123 | 0.212 | 0.162 | 54 |
| 95 | 0 | 0.737 | 0.074 | 0.094 | 0.081 | 0.210 | 0.199 | 41 |

GPo - Germination potential, GPe - Germination percentage, PL - Plumule length, RL - Radicle length, PFW - Plumule fresh weight, RFW - Radicle fresh weight, *D* - Average of fuzzy membership values.
