# Supplementary material for: Evaluation of cold tolerance in sorghum germplasm from the Chishui River Basin in China: insights from germination, field trials, and physiological assays
Source: Front Plant Sci. 2025 Sep 2;16:1630271. doi: 10.3389/fpls.2025.1630271 (PMC12436481; doi:10.3389/fpls.2025.1630271)
Supplement: Supplementary file 5 [file Table5.doc]

Supplementary Table 5. Membership function analysis of sorghum germination characteristics under 15℃ cold stress treatment.

| Materia | GPo | GPe | PL | RL | PFW | RFW | *D* | Sorting |
| --- | --- | --- | --- | --- | --- | --- | --- | --- |
| 1 | 0.203 | 0.361 | 0.326 | 0.181 | 0.281 | 0.240 | 0.265 | 54 |
| 2 | 0.576 | 0.501 | 0.230 | 0.182 | 0.534 | 0.717 | 0.457 | 23 |
| 3 | 0.503 | 0.601 | 0.221 | 0.230 | 0.469 | 0.974 | 0.500 | 17 |
| 4 | 0.221 | 0.415 | 0.365 | 0.162 | 0.309 | 0.488 | 0.327 | 41 |
| 5 | 0.679 | 0.238 | 0.149 | 0.077 | 0.118 | 0.687 | 0.324 | 42 |
| 6 | 0.222 | 0.466 | 0.246 | 0.365 | 0.150 | 0.577 | 0.338 | 39 |
| 7 | 0.602 | 0.404 | 0.326 | 0.283 | 0.229 | 0.741 | 0.431 | 29 |
| 8 | 0.440 | 0.513 | 0.201 | 0.340 | 0.166 | 0.758 | 0.403 | 32 |
| 9 | 0.441 | 0.553 | 0.366 | 0.474 | 0.270 | 0.604 | 0.451 | 24 |
| 10 | 0.532 | 0.209 | 0.319 | 0.542 | 0.471 | 0.594 | 0.444 | 27 |
| 11 | 0.059 | 0.460 | 0.510 | 0.191 | 0.179 | 0.479 | 0.313 | 46 |
| 12 | 0.710 | 0.706 | 0.775 | 0.606 | 0.546 | 0.867 | 0.702 | 2 |
| 13 | 0.430 | 0.425 | 0.412 | 0.443 | 0.438 | 0.891 | 0.507 | 16 |
| 14 | 0.261 | 0.191 | 0.229 | 0.089 | 0.249 | 0.605 | 0.271 | 53 |
| 15 | 0.243 | 0.462 | 0.248 | 0.418 | 0.383 | 0.383 | 0.356 | 35 |
| 16 | 0.326 | 0.205 | 0.062 | 0.133 | 0.350 | 0.235 | 0.218 | 60 |
| 17 | 0.053 | 0.078 | 0.038 | 0.096 | 0.148 | 0.490 | 0.150 | 67 |
| 18 | 0.047 | 0.499 | 0.267 | 0.647 | 0.241 | (0.000) | 0.284 | 49 |
| 19 | 0.387 | 0.645 | 0.350 | 0.502 | 0.605 | 0.901 | 0.565 | 9 |
| 20 | 0.053 | 0.529 | 0.567 | 0.208 | 0.274 | 0.223 | 0.309 | 47 |
| 21 | 0.415 | 0.950 | 1.000 | 0.496 | 0.382 | 0.981 | 0.704 | 1 |
| 22 | 0.609 | 0.406 | 0.465 | 0.652 | 1.000 | 0.722 | 0.642 | 4 |
| 23 | 0.157 | 0.643 | 0.815 | 0.427 | 0.715 | 0.578 | 0.556 | 10 |
| 24 | 0.230 | 0.665 | 0.512 | 0.516 | 0.407 | 0.775 | 0.517 | 14 |
| 25 | 0.504 | 0.478 | 0.588 | 0.581 | 0.685 | 0.809 | 0.608 | 5 |
| 26 | 0.450 | 0.484 | 0.767 | 0.481 | 0.246 | 0.204 | 0.439 | 28 |
| 27 | 0.356 | 0.285 | 0.272 | 0.366 | 0.167 | 0.237 | 0.281 | 50 |
| 28 | 0.276 | 0.213 | 0.330 | 0.187 | 0.230 | 0.209 | 0.241 | 59 |
| 30 | 0.457 | 0.619 | 0.546 | 0.341 | 0.489 | 0.423 | 0.479 | 20 |
| 32 | 0.339 | 0.610 | 0.441 | 0.291 | 0.453 | 0.666 | 0.467 | 22 |
| 38 | 0.710 | 0.680 | 0.514 | 0.234 | 0.385 | 0.621 | 0.524 | 13 |
| 39 | 0.305 | 0.983 | 0.613 | 0.498 | 0.579 | 0.585 | 0.594 | 6 |
| 40 | 0.154 | 1.000 | 0.513 | 0.363 | 0.650 | 0.369 | 0.508 | 15 |
| 41 | 0.000 | 0.566 | 0.486 | 1.000 | 0.586 | 0.293 | 0.488 | 19 |
| 42 | 0.155 | 0.748 | 0.527 | 0.244 | 0.373 | 0.621 | 0.445 | 26 |
| 43 | 0.502 | 0.825 | 0.494 | 0.425 | 0.781 | 0.519 | 0.591 | 7 |
| 44 | 0.206 | 0.069 | 0.030 | 0.103 | 0.389 | 0.249 | 0.174 | 64 |
| 45 | 0.891 | 0.708 | 0.516 | 0.347 | 0.609 | 0.953 | 0.670 | 3 |
| 46 | 0.433 | 0.171 | 0.039 | 0.149 | 0.200 | 0.927 | 0.320 | 44 |
| 47 | 0.127 | 0.110 | 0.224 | 0.120 | 0.048 | 0.260 | 0.148 | 68 |
| 48 | 0.590 | 0.198 | 0.203 | 0.002 | 0.040 | 0.454 | 0.248 | 57 |
| 49 | 0.599 | 0.238 | 0.176 | 0.113 | 0.119 | 0.771 | 0.336 | 40 |
| 50 | 0.000 | 0.149 | 0.051 | 0.044 | 0.000 | 0.812 | 0.176 | 63 |
| 51 | 0.162 | 0.109 | 0.138 | 0.028 | 0.017 | 0.531 | 0.164 | 65 |
| 52 | 0.149 | 0.112 | 0.258 | 0.051 | 0.017 | 0.346 | 0.155 | 66 |
| 53 | 0.332 | 0.768 | 0.431 | 0.463 | 0.676 | 0.509 | 0.530 | 12 |
| 67 | 0.163 | 0.216 | 0.057 | 0.105 | 0.061 | 0.165 | 0.128 | 70 |
| 68 | 0.099 | 0.496 | 0.323 | 0.106 | 0.253 | 0.508 | 0.298 | 48 |
| 73 | 0.060 | 0.243 | 0.115 | 0.230 | 0.243 | 0.756 | 0.274 | 52 |
| 74 | 0.471 | 0.429 | 0.414 | 0.162 | 0.110 | 0.942 | 0.421 | 31 |
| 75 | 0.701 | 0.507 | 0.370 | 0.181 | 0.736 | 1.000 | 0.582 | 8 |
| 76 | 0.284 | 0.634 | 0.683 | 0.286 | 0.266 | 0.412 | 0.428 | 30 |
| 77 | 0.387 | 0.173 | 0.127 | 0.119 | 0.031 | 0.449 | 0.214 | 61 |
| 78 | 0.200 | 0.135 | 0.119 | 0.088 | 0.097 | 0.248 | 0.148 | 69 |
| 79 | 0.190 | 0.107 | 0.001 | 0.021 | 0.323 | 0.067 | 0.118 | 71 |
| 80 | 0.348 | 0.096 | 0.106 | 0.159 | 0.559 | 0.843 | 0.352 | 36 |
| 81 | 0.697 | 0.416 | 0.544 | 0.248 | 0.636 | 0.453 | 0.499 | 18 |
| 82 | 0.573 | 0.252 | 0.229 | 0.094 | 0.145 | 0.637 | 0.322 | 43 |
| 83 | 0.908 | 0.344 | 0.280 | 0.140 | 0.207 | 0.829 | 0.451 | 25 |
| 84 | 0.463 | 0.184 | 0.202 | 0.124 | 0.328 | 0.237 | 0.256 | 55 |
| 85 | 0.431 | 0.166 | 0.189 | 0.276 | 0.745 | 0.531 | 0.390 | 33 |
| 86 | 0.159 | 0.164 | 0.077 | 0.000 | 0.006 | 0.796 | 0.200 | 62 |
| 87 | 0.602 | 0.131 | 0.202 | 0.027 | 0.242 | 0.842 | 0.341 | 37 |
| 88 | 0.413 | 0.000 | 0.000 | 0.028 | 0.019 | 0.984 | 0.241 | 58 |
| 89 | 0.412 | 0.252 | 0.308 | 0.274 | 0.196 | 0.595 | 0.339 | 38 |
| 90 | 0.000 | 0.247 | 0.332 | 0.151 | 0.179 | 0.595 | 0.251 | 56 |
| 91 | 0.533 | 0.188 | 0.138 | 0.167 | 0.133 | 0.747 | 0.318 | 45 |
| 92 | 0.441 | 0.243 | 0.255 | 0.109 | 0.523 | 0.569 | 0.357 | 34 |
| 93 | 0.676 | 0.189 | 0.198 | 0.287 | 0.689 | 0.807 | 0.474 | 21 |
| 94 | 0.050 | 0.204 | 0.193 | 0.228 | 0.362 | 0.640 | 0.279 | 51 |
| 95 | 0.595 | 0.462 | 0.136 | 0.686 | 0.501 | 0.890 | 0.545 | 11 |

GPo - Germination potential, GPe - Germination percentage, PL - Plumule length, RL - Radicle length, PFW - Plumule fresh weight, RFW - Radicle fresh weight, *D* - Average of fuzzy membership values.
