# Supplementary material for: Evaluation of cold tolerance in sorghum germplasm from the Chishui River Basin in China: insights from germination, field trials, and physiological assays
Source: Front Plant Sci. 2025 Sep 2;16:1630271. doi: 10.3389/fpls.2025.1630271 (PMC12436481; doi:10.3389/fpls.2025.1630271)
Supplement: Supplementary file 6 [file Table6.doc]

Supplemtntary Table 6. Variance analysis of measured value between physiological index.

| Treatment | Parameters | RWC  （%） | PMP  （%） | CHL  (mg/g) | SOD  (Unit/g) | POD  (Unit/g) | MDA  (nmol/g ) | SP  (mg/g) | SS  (mg/g) |
| --- | --- | --- | --- | --- | --- | --- | --- | --- | --- |
| Cold | Max | 93.81 | 20.93 | 1.93 | 297.38 | 386.00 | 34.42 | 173.43 | 41.29 |
|  | Min | 85.37 | 15.02 | 1.66 | 101.55 | 200.00 | 16.05 | 156.04 | 19.16 |
|  | Average | 91.41 | 18.40 | 1.82 | 202.28 | 292.00 | 21.31 | 165.82 | 25.88 |
|  | SD | 2.19 | 1.59 | 0.07 | 69.79 | 63.61 | 5.11 | 4.48 | 5.65 |
|  | CV | 2.40 | 8.64 | 3.85 | 34.50 | 21.78 | 23.98 | 2.70 | 21.83 |
| CK | Max | 96.63 | 19.06 | 2.08 | 196.89 | 224.00 | 21.13 | 166.13 | 22.34 |
|  | Min | 87.93 | 13.56 | 1.86 | 76.66 | 152.00 | 12.38 | 140.54 | 10.26 |
|  | Average | 94.23 | 16.02 | 1.95 | 131.42 | 184.41 | 15.31 | 156.00 | 15.72 |
|  | SD | 2.38 | 1.55 | 0.06 | 45.59 | 16.11 | 2.34 | 6.07 | 3.96 |
|  | CV | 2.52 | 9.68 | 3.08 | 34.69 | 8.74 | 15.28 | 3.89 | 25.19 |
| DCC | Average | -2.82 | 2.38 | -0.13 | 70.86 | 107.59 | 6.00 | 9.82 | 10.16 |
|  | *t* Value | -4.517 | 5.552 | -7.021 | 4.417^**^ | 8.520^**^ | 5.550^*^ | 6.766 | 7.651 |

DCC-cold change than control, Max - Maximum value, Min - Minimum value, SD - Standard deviation, CV - Coefficient of variation, RWC-leaf relative water conent, PMP-plasma membrane permeability, CHL-chlorophyll content, SOD-catalase, POD-peroxidase, MDA-malondialdehyde, SP-soluble protein, SS-soluble sugar, *t* value-paired t test between treatment and control，** ,* indicate t test amount to significant.
