# Supplementary material for: Evaluation of cold tolerance in sorghum germplasm from the Chishui River Basin in China: insights from germination, field trials, and physiological assays
Source: Front Plant Sci. 2025 Sep 2;16:1630271. doi: 10.3389/fpls.2025.1630271 (PMC12436481; doi:10.3389/fpls.2025.1630271)
Supplement: Supplementary file 7 [file Table7.doc]

Supplemtntary Table 7. Correlation analysis of chilling tolerance indices of physiological indicators.

| Index | RWC | PMP | CHL | SOD | POD | MDA | SP | SS |
| --- | --- | --- | --- | --- | --- | --- | --- | --- |
| RWC | 1 |  |  |  |  |  |  |  |
| PMP | -0.259 | 1 |  |  |  |  |  |  |
| CHL | 0.282 | -434* | 1 |  |  |  |  |  |
| SOD | 0.534** | -0.719** | 0.496** | 1 |  |  |  |  |
| POD | 0.553** | -0.756** | 0.696** | 0.795** | 1 |  |  |  |
| MDA | -0.287 | 0.523** | -0.503** | -0.762** | -0.738** | 1 |  |  |
| SP | -0.087 | -0.244 | 0.305 | 0.373 | 0.264 | -0.398* | 1 |  |
| SS | 0.335 | -0.521** | 0.708** | 0.586** | 0.675** | -0.527** | 0.179 | 1 |

RWC-leaf relative water conent, PMP-plasma membrane permeability, CHL-chlorophyll content, SOD-catalase, POD-peroxidase, MDA-malondialdehyde, SP-soluble protein, SS-soluble sugar. ** and * indicate significant level of *p* < 0.01 and *p* < 0.05.
