# Supplementary material for: Evaluation of cold tolerance in sorghum germplasm from the Chishui River Basin in China: insights from germination, field trials, and physiological assays
Source: Front Plant Sci. 2025 Sep 2;16:1630271. doi: 10.3389/fpls.2025.1630271 (PMC12436481; doi:10.3389/fpls.2025.1630271)
Supplement: Supplementary file 8 [file DataSheet1.pdf]

Supplementary Figure 1

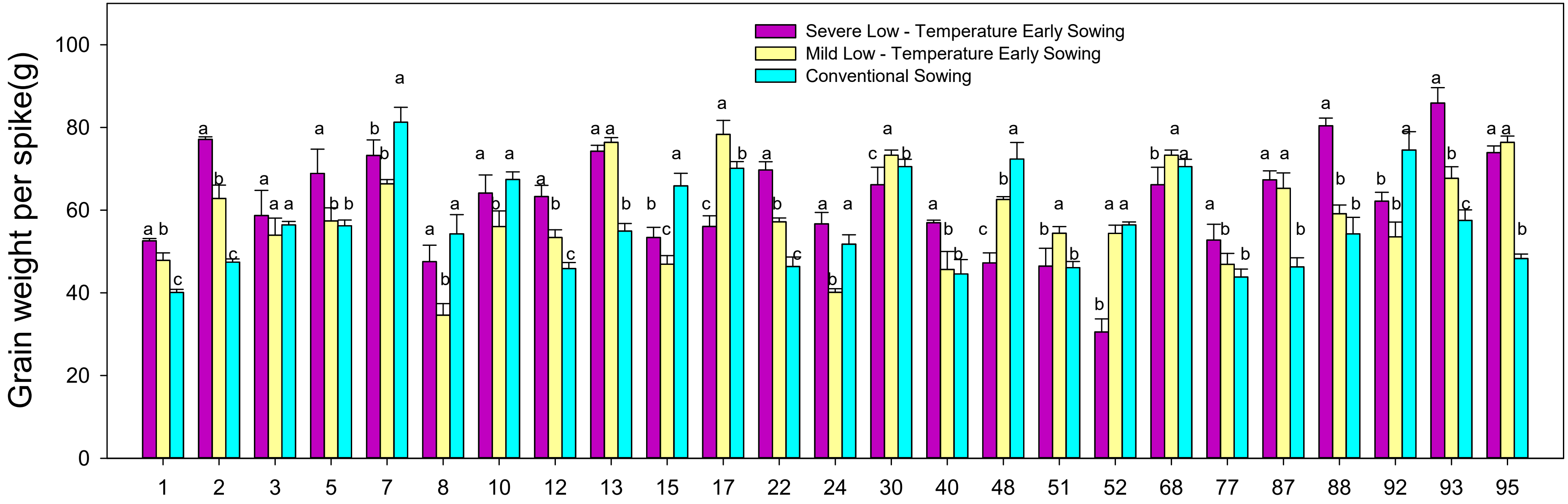

Supplementary Figure 1 Effect of early Sowing on the of 25 sorghum germplasm accessions. Data are the mean values of three replicates. Significant differences ($P < 0.05$, Duncan's Test) among three sowing dates of the same sorghum germplasm, indicated by different letters.
