# Supplementary material for: Evaluation of cold tolerance in sorghum germplasm from the Chishui River Basin in China: insights from germination, field trials, and physiological assays
Source: Front Plant Sci. 2025 Sep 2;16:1630271. doi: 10.3389/fpls.2025.1630271 (PMC12436481; doi:10.3389/fpls.2025.1630271)
Supplement: Supplementary file 9 [file DataSheet2.pdf]

Supplementary Figure 2

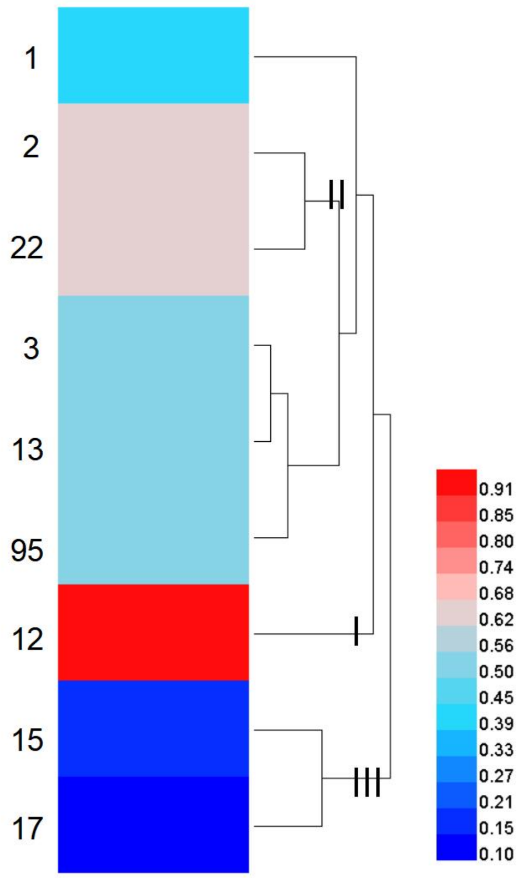

Supplementary Figure 2 Cluster analysis of 9 sorghum germplasms. I represents the cold-tolerant type. II represents the moderately cold-tolerant type. III represents the moderately sensitive type. IV represents the temperature-sensitive type.The color bar on the right side indicates the range of Z-scores used for the heatmap. The color scale ranges from blue (low Z-score) to red (high Z-score).
